# CT perfusion imaging in aneurysmal subarachnoid hemorrhage. State of the art

**DOI:** 10.3389/fradi.2024.1445676

**Published:** 2024-10-07

**Authors:** Valentina Elisabetta Lolli, Adrien Guenego, Niloufar Sadeghi, Lise Jodaitis, Boris Lubicz, Fabio Silvio Taccone, Elisa Gouvea Bogossian

**Affiliations:** ^1^ Radiology Department, Hôpital Erasme- H.U.B., Brussels, Belgium; ^2^ Interventional Neuroradiology Department, Hôpital Erasme- H.U.B., Brussels, Belgium; ^3^ Neurology Department, Hôpital Erasme- H.U.B., Brussels, Belgium; ^4^ Intensive Care Department, Hôpital Erasme- H.U.B., Brussels, Belgium

**Keywords:** delayed cerebral ischemia, aneurysmal subarachnoid hemorrhage, cerebral vasospasm, hypoperfusion, computed tomography perfusion

## Abstract

CT perfusion (CTP) images can be easily and rapidly obtained on all modern CT scanners and have become part of the routine imaging protocol of patients with aneurysmal subarachnoid haemorrhage (aSAH). There is a growing body of evidence supporting the use of CTP imaging in these patients, however, there are significant differences in the software packages and methods of analysing CTP. In. addition, no quantitative threshold values for tissue at risk (TAR) have been validated in this patients’ population. Here we discuss the contribution of the technique in the identification of patients at risk of aSAH-related delayed cerebral ischemia (DCI) and in the assessment of the response to endovascular rescue therapy (ERT). We also address the limitations and pitfalls of automated CTP postprocessing that are specific to aSAH patients as compared to acute ischemic stroke (AIS).

## 1 Introduction

CT perfusion (CTP) represents the imaging modality of choice for patients’ selection in acute ischemic stroke (AIS) triage, and there is a growing body of evidence supporting its use in patients with aneurysmal subarachnoid haemorrhage (aSAH). CTP images can be easily and rapidly obtained on all modern CT scanners with very limited user input and easy accessibility of the automatically generated color-coded CTP summary maps. However, as highlighted by Chung et al. (1) in a very informative review on the use of CTP in AIS, the easy accessibility of CTP images overshadows the complexity of the underlying computational postprocessing workflow and may lead to overconfidence in interpretation thereby favoring diagnostic errors. This is especially true in aSAH, due to the high incidence of associated intraparenchymal hemorrhage and intracranial devices, which represent an additional source of artefacts and potential error.

Several studies have investigated the contribution of CTP in this specific patients’ population and have yielded conflicting results. In addition, there are currently no definite quantitative threshold values for defining tissue at risk (TAR) in aSAH patients.

In this work, we discuss the contribution of the technique in the identification of aSAH-related delayed cerebral ischemia (DCI) and in the assessment of the response to endovascular rescue therapy (ERT). We discuss CTP acquisition and post-processing, and we address the limitations and pitfalls of the technique that are specific to aSAH patients as compared to AIS.

## 2 Discussion

### 2.1 Aneurysmal subarachnoid hemorrhage (aSAH)

aSAH is rare, accounting for only 5% of all strokes (2). This condition, however, carries an exceptionally high disease-specific burden, affecting younger adults as compared to AIS and accounting for 20% of all stroke-related years of life lost before age 60 (3). According to a recent meta-analysis including 75 studies and 8,176 patients, there has been a decrease in the incidence of aSAH from 10.2 per 100, 000 person per year in 1980 to 6.1 in 2010 (4), along with the decrease in blood pressure and smoking prevalence. Similarly, advances in diagnosis and management strategies of ruptured intracranial aneurysms and SAH led to a 17% decrease of the case fatality of aSAH from 1995 to 2007 (5). In more recent years, conversely, mortality has remained unchanged, ranging from 13.7% in 2006 to 13.1% in 2018 in the United States (6). A similar trend was reported by our group: in a retrospective study aimed at investigating the time course of mortality and neurological outcome in 353 poor grade non-traumatic SAH patients between 2004 and 2018, the authors observed no significant reduction of mortality rates over the years and no changes in the incidence of unfavorable neurological outcome, which remained high (7).

Despite the intense research efforts in the field, there is still a lack of in-depth understanding and effective treatment of the processes responsible for secondary brain injury after aSAH. To date, most aSAH survivors experience long-term disability and/or cognitive dysfunction (8, 9), with a loss of productive life-years similar to AIS on a community level (5).

### 2.2 Delayed cerebral ischemia (DCI): definition and pathophysiology. Role of cerebral vasospasm

DCI occurs in 20%–30% of patients with aSAH and represents one of the most important causes of poor neurological outcome in this patients’ population despite aggressive treatment (10). Diagnostic criteria of DCI include the following: the appearance of focal (i.e., hemiparesis, aphasia, hemianopsia or neglect) or global (i.e., 2-point decrease in the Glasgow Coma Scale, GCS) neurological impairment, lasting at least 1 h, and/or cerebral ischemia on brain imaging, unrelated to the initial bleeding or surgical procedures (11). Cerebral vasospasm—the narrowing of cerebral arteries occurring a few days after aSAH - is recognized as a major contributor in the pathogenesis of DCI. It affects up to 70% of patients with aSAH and is symptomatic in nearly half of cases (12). Cerebral vasospasm typically begins after days 3 to 4 following aSAH and reaches a peak at days 7–10. In patients who survive, it spontaneously resolves after 3 to 4 weeks (13). This phenomenon is thought to result from prolonged smooth muscle cells contraction mediated by subarachnoid blood. Cerebrospinal fluid (CSF) oxyhemoglobin in particular, seems to play a major role in the cascade of events that take place after aSAH and that ultimately cause vasoconstriction (14, 15). A relationship between the volume of blood in the subarachnoid space and the severity of vasospasm has been demonstrated in both animal and human patients (16–18). Fisher et al. (16) were the first to investigate the relationship of the amount and distribution of subarachnoid blood seen on CT to the later development of cerebral vasospasm and reported an almost exact correspondence between the site of the major subarachnoid blood clots and the location of severe vasospasm. Accordingly, increasing grades 1 to 4 of the so-called modified “Fisher scale”, which takes into account thick cisternal and ventricular blood, are associated with a progressive risk of developing vasospasm (19). In line with these observations, experimental research has shown that targeting CSF oxyhaemoglobin reduces DCI and improves neurological outcome after SAH in animal models (20).

Large-arteries narrowing with consequent decreased cerebral blood flow (CBF) occurring a few days after aneurysm rupture may result in cerebral ischemia (21). Different studies have investigated the relationship between vasospasm on CT angiography (CTA) and CTP findings and there is some discrepancy in their results. Numerous works have shown an association between vasospasm and the presence of a perfusion deficit in the corresponding parenchymal territory (22–26). According to Sanelli et al. (25), aSAH patients with > 50% angiographic narrowing of a given artery tend to have a perfusion deficit with reduced CBF. Dankbaar et al. (27) investigated the effects of vasospasm on cerebral perfusion in a population of 40 aSAH patients and reported a correlation between the severity of vasospasm and the degree of the perfusion deficit: in 15 out of 23 (65%) patients with moderate to severe vasospasm, the parenchymal territory supplied by the most severely affected artery corresponded with the least perfused region. Contradictory results, however, have also been reported. Many patients with large- or medium-sized arteries vasospasm do not have any perfusion deficit (28, 29). Also, many aSAH patients with cerebral infarctions do not have evidence of vasospasm involving the large- or medium-sized vessels (30, 31). These conflicting observations have suggested that cerebral vasospasm does affect cerebral perfusion but is *per se* not sufficient to cause DCI.

The causal framework of DCI seems indeed to include other factors than cerebral vasospasm. These include early brain injury (<72 h) due to inflammation, oxidative stress and apoptosis; microcirculatory disturbances with loss of autoregulation; cortical spreading depolarization (32, 33); and microthrombosis (34–36) - which occur independently from large- or medium arteries narrowing (37). These seem to occur as a continuum triggered by the initial bleeding, which ultimately leads to cellular death and blood-brain barrier disruption.

### 2.3 CT angiography (CTA)

CTA is a rapid and non-invasive technique that has proven to be highly sensitive, specific, and accurate as compared to digital subtraction angiography (DSA) - which represents the reference standard—in detecting severe cerebral vasospasm in proximal arterial locations, while being less accurate for detecting mild and moderate distal vasospasm (38). The technique yields a very high negative predictive value, the lack of large-arteries vasospasm on day 8 or later after aSAH indicating a very low risk of DCI (21). Angiographic assessment of vasospasm is both subjective and semiquantitative, relying on the comparison of the size of the cerebral arteries to an internal standard, such as the distal internal carotid artery or to a prior baseline study. There is a general consensus on defining severe vasospasm as a greater than 50% reduction in the vessel diameter, moderate vasospasm as a 25%–50% reduction and mild vasospasm as a less than 25% reduction in the vessel lumen [see e.g., (11, 28, 39)].

### 2.4 CT perfusion (CTP) imaging

#### 2.4.1 CT perfusion (CTP): technique

CTP is a functional imaging technique that allows to quantify blood flow through the brain parenchyma by using an exogenous intravascular tracer and serial imaging during its first circulation through the brain capillary bed. According to the most recent American College of Radiology recommendations (40), a minimum volume of 30 ml of an iodinated contrast medium is injected at a rate of at least 4 ml/s through a large-bore peripheral IV catheter; after a 5–7 s delay, images are acquired at 70–90 kVp and 100–200 mAs every 1–3 s for at least 50–60 s. In children, smaller injection rates and smaller volumes of contrast are recommended (41). Modern CT scanners allow for whole-brain coverage with this technique.

Rapid imaging of the brain is performed repeatedly as the contrast bolus flows through the brain, and the relative increase, peak and then decrease in density, measured in Hounsfield units (HU), allow a time-attenuation curve (TAC) to be derived for each voxel. Tracer kinetic models are then used to estimate hemodynamic parameters from the 4-dimensional data set of TACs. The most commonly used parameters are:

• Cerebral blood volume (CBV): the total volume of flowing blood per unit of brain mass (ml/min/100 g).
• Cerebral blood flow (CBF): the flow rate of blood through a unit of brain mass (ml/min/100 g). Measurement of CBF is usually not quantitative but rather obtained by normalization to an unaffected region of the brain. Accordingly, CBF is expressed as a percentage as compared to the reference ROI and is referred to as relative CBF (rCBF).
• Mean transit time (MTT): the mean time for blood to perfuse a region of tissue (sec). MTT is related to CBF and CBV by the central volume principle: MTT = CBV/CBF.
• Time to peak (TTP): the time to the maximum point of the time-signal curve (sec). It represents the time at which the maximum change in tracer concentration occurs after the passage of the bolus (42, 43).
• Time to maximum (TMax): similar to TTP, TMax reflects the time from the start of the scan until the maximum peak of contrast material in each voxel (sec).
• Time to start (TTS): the time from arterial enhancement to tissue enhancement (sec).
• Time to drain (TTD): the time taken for contrast medium washout, from maximum enhancement to a defined low threshold (sec).

**Figure 1:**
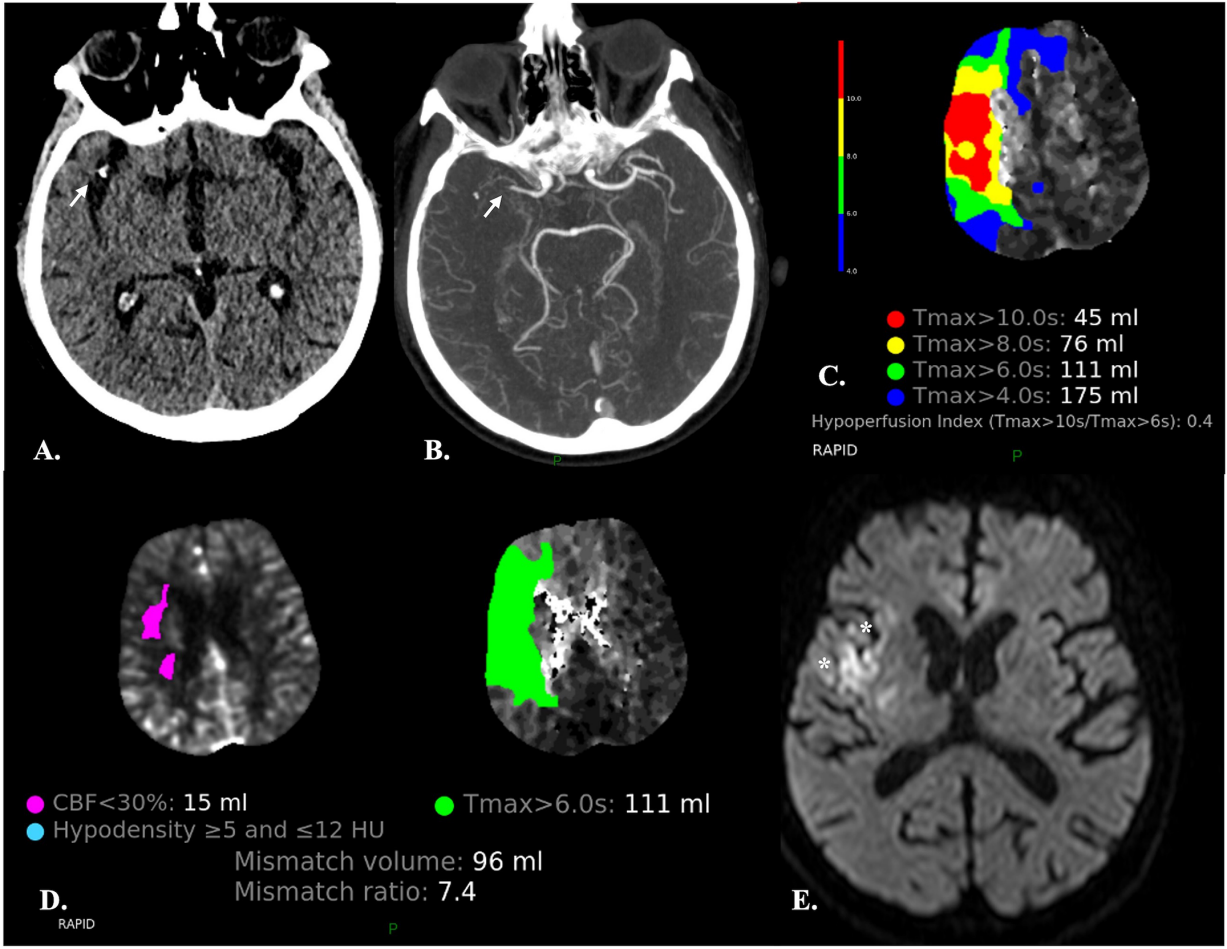
Automatic segmentation of the brain parenchyma into normal tissue, tissue at risk (TAR) and non-viable tissue (NVT) by the CTP RAPID software in a 67-year-old male with abrupt onset left-sided hemiparesis. **(A,B)** NECT **(A)** and CTA **(B)** demonstrate occlusion of the M1 segment of the right MCA (*arrows)* and hypodensity of the insular cortex (not shown) resulting in an ASPECTS score of 9. **(C)** The time-to-maximum (TMax) CTP colour map shows different TMax thresholds (10, 8, 6 and 4 s) for TAR estimation: the lower the threshold, the larger the TAR volume. The hypoperfusion intensity ratio (HIR) (i.e., the Tmax > 10 s/Tmax >6 s ratio) is also provided. A low HIR is considered a predictor of slow ischemic core growth. Conversely, a high HIR is associated with a rapid growth of the infarct core (44). **(D)** Automated quantification of TAR and NVT and calculation of mismatch ratios are provided for treatment decision. Automated identification of areas with density varying between ≥5 and ≤12 HU, presumably consistent with acute ischemic lesions, is also carried out. **(E)** Diffusion-weighted MRI obtained two days after mechanical thrombectomy showed a very limited area of ischemia (*) as compared to the previously observed Tmax > 6 s, indicating that reperfusion has been effective in salvaging the ischemic penumbra.

Each of these hemodynamic parameters is typically displayed as a color map of the brain, with a sequential scale representing the quantitative data values in each voxel. Automated postprocessing software further segment the brain parenchyma into normal tissue, tissue at risk (TAR), and non-viable tissue (NVT) based on quantitative or comparative parameter thresholds. For clinical use, indeed, the aim of CTP is to quantify tissue that is irreversibly infarcted (the *infarct core*, i.e., the NVT) and tissue that is hypoperfused and is thus considered at risk of infarction (the *penumbra,* i.e., the TAR) in the absence of reperfusion. Quantitative volume measurements of TAR and NVT are provided, along with prognostic summary maps and mismatch ratios that can be used for treatment decision (Figure 1).

**Figure 2:**
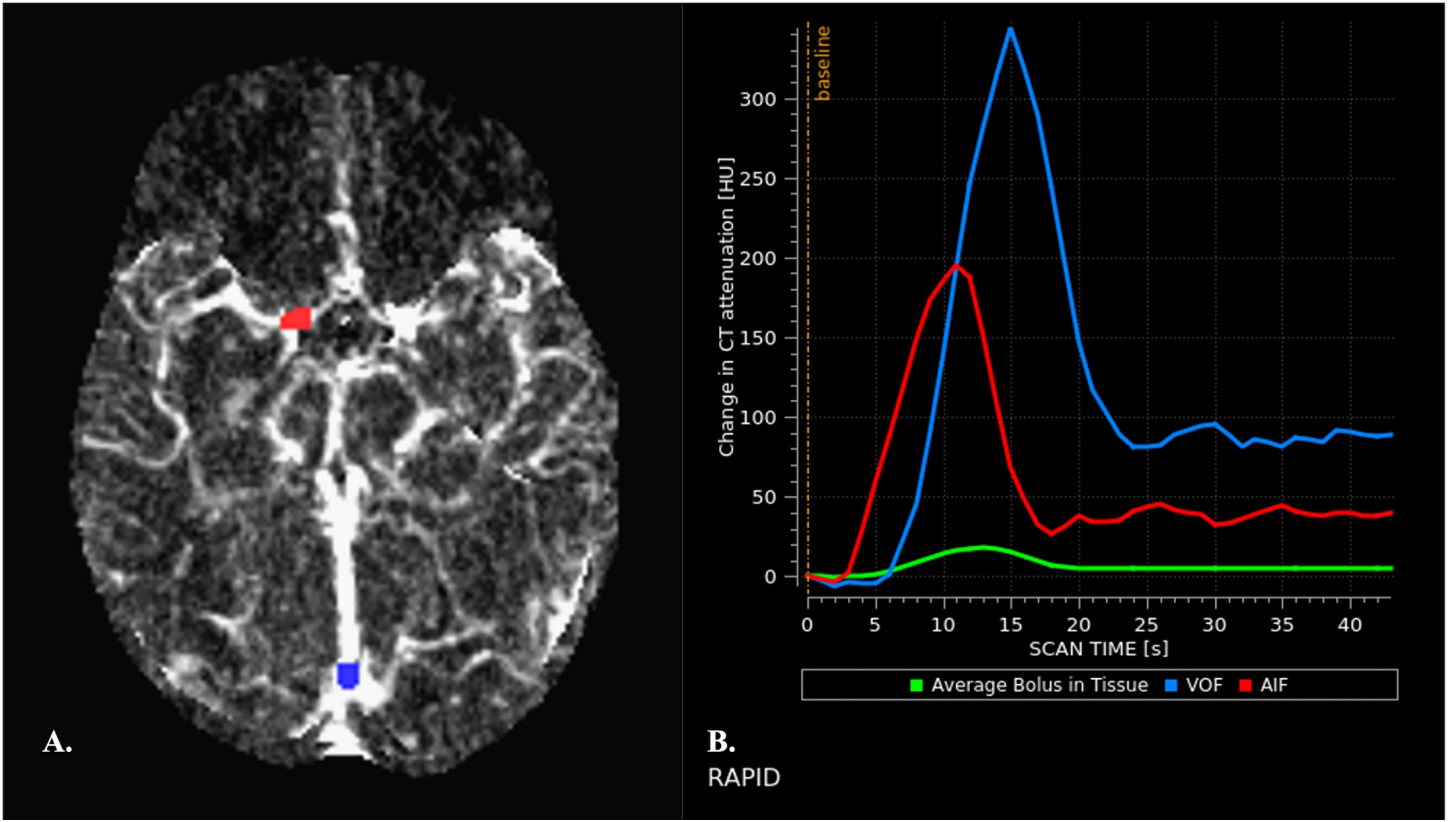
Automated arterial and venous ROIs placement **(A)** and generation of arterial and venous TACs **(B) (A)** appropriate placement of arterial and venous ROIs is crucial for obtaining valid perfusion maps. Arterial and venous ROIs are automatically placed by the software on CTP source images and ROI maps are then displayed, allowing assessment of accuracy before interpretation. Arterial ROIs should be placed is an artery that travels perpendicular to the axial plane to reduce partial volume effects (PVEs), in the unaffected cerebral hemisphere (45). The terminal internal carotid artery, proximal middle cerebral artery, and anterior cerebral artery are commonly used. Arteries of the posterior circulation are conversely avoided because of the relative physiological delay in bolus arrival in these anatomic territories (46). Despite appropriate placement of AIF ROI, the area under the curve (AUC) of the AIF may be underestimated due to PVEs. Therefore, the AUC AIF needs to be normalized with respect to the AUC of the VOF as measured in a large vein. Venous ROIs are commonly placed in the superior sagittal sinus or the torcular. It is indeed assumed that the VOF is unhampered by PVEs (47). **(B)** Normal AIF and VOF curves are shown. They display characteristics features: (1) a baseline interval, that precedes the arrival of the bolus of contrast medium. The duration of the baseline interval before arrival of the contrast material provides an indirect index of cardiac function in each patient. (2) a subsequent upslope interval that indicates rapid increase of attenuation, corresponding to the arrival of the bolus of contrast material into the ROI. (3) a rapid decrease in attenuation, as the contrast bolus leaves the ROI, with return to a near steady-state attenuation. (4) a “plateau” interval that reflects the arrival of recirculated contrast material (48). Width of the curve reflects the speed of contrast material bolus infusion. The VOF peak is typically higher than the AIF peak and is delayed by 1–2 s.

The color-coded CTP maps available for interpretation on PACS or mobiles are the result of complex mathematical computation. This process requires both an arterial input function (AIF) and a venous output function (VOF), that are obtained by defining arterial and venous ROIs in the CTP source images (Figure 2). This post-processing step is typically performed automatically by the software and is crucial for obtaining valid CTP maps. Incorrect ROIs placement may indeed result in inaccurate estimation of perfusion parameter. Therefore, it is imperative to examine ROI maps to ensure appropriateness of automatically placed ROIs before interpretation (45, 47, 49).

Numerous factors contribute to the shape of CTP TACs, which include the characteristics of the injection, variability in cardiac output and in the upstream extra- or intracranial arterial flow to the brain area of interest (1, 50, 51). The expected temporal profile of arrival, transit and washout of the bolus of contrast material may be altered in several conditions, that result in delay, or dispersion, of the contrast bolus, with consequent degradation of the native TACs within individual voxels (43).

Among the different methods available to calculate the perfusion parameters of interest, deconvolution has the advantage of minimizing the effect of the AIF on native TACs, thus allowing to isolate intrinsic tissue-level hemodynamic properties—referred to as “the residue function”. The software that do not use deconvolution, conversely, have been proven more prone to pitfalls related to delay, or dispersion, of the contrast bolus (43). Another important practical advantage of deconvolution is that the technique allows for much lower injection rates as compared to other techniques of analysis such as maximum slope (3–4 ml/s vs. 8–10 ml/s); therefore, deconvolution is widely used in clinical practice. Deconvolution provides the CBF, MTT and TMax. Other perfusion parameters can be estimated directly from the parenchymal TACs, without deconvolution: these include CBV as well as parameters that provide similar information to MTT [full width at half maximum (FWHM), first moment of transit (FMT), bolus arrival time (BAT), TTP, and bolus end time (BET)], or to CBF [maximal slope of the time–concentration curve (MS)], or similar to CBF and CBV [maximal value of the time–concentration curve (Cmax)] (43). However, as highlighted by Leiva-Salinas et al. (43) and mentioned here-above, such parameters are subject to inaccuracies related to the effects of the AIF on native TACs. These used to be useful when performing deconvolution was exceedingly time-consuming, but this is no longer the case with modern software.

**Figure 3:**
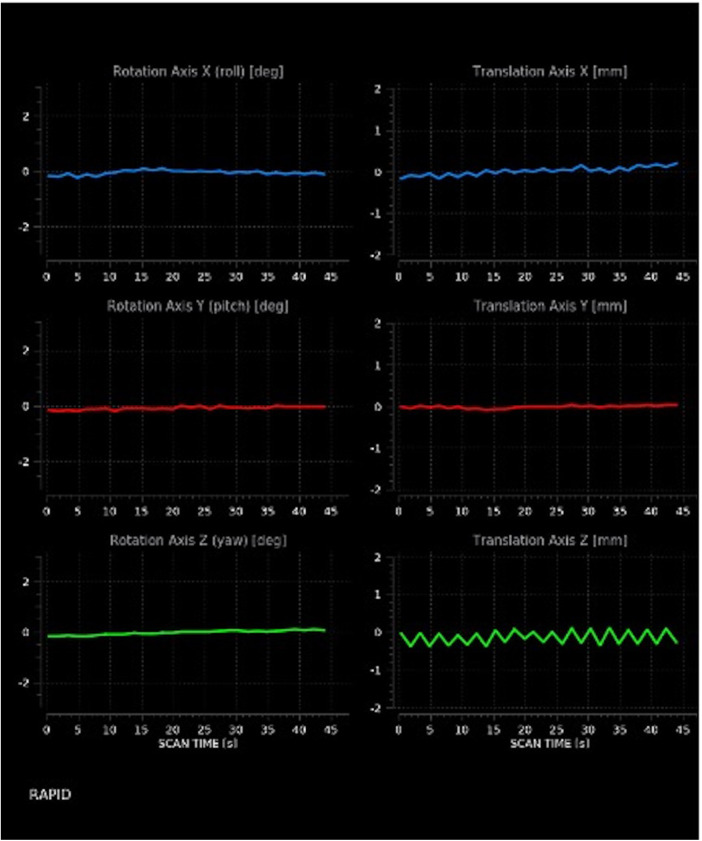
Motion estimates. The image discloses moderate translation on the x- and z-axes, as shown by the indented rather than straight appearance of corresponding lines.

Before deconvolution of CTP raw data, an important pre-processing step automatically performed by the software is represented by realignment. Because of the dynamic acquisition (i.e., sequential CT acquisitions during the intravenous administration of a bolus of iodinated contrast material), even slight motion can degrade CTP raw data, with consequent decreased reliability of results. To address this issue, most commercially available software packages apply built-in motion correction algorithms which can allow generation of valid TACs. The 6 rigid motion parameters are displayed in line plots, that the radiologist needs to check to make sure that motion correction has been effectively carried out (Figure 3).

#### 2.4.2 CT perfusion (CTP) in acute ischemic stroke (AIS)

CTP represents the imaging modality of choice to evaluate the presence of cerebral TAR in patients with AIS and the accuracy of the technique has launched a shift to imaging-based selection of potential candidates for endovascular treatment. CTP indeed allows to identify patients who may benefit from reperfusion therapy beyond the conventionally accepted time window or in whom time of symptom onset is unknown (52–54). Commonly used CTP thresholds include rCBF < 30% for NVT and TMax > 6 s for TAR (52, 53, 55). These were specifically designed for AIS. Of note, they slightly underestimate the core infarct volume, thus favoring inclusion for treatment (56, 57).

#### 2.4.3 Ct perfusion (CTP) in aneurysmal subarachnoid hemorrhage (aSAH)

**Figure 4:**
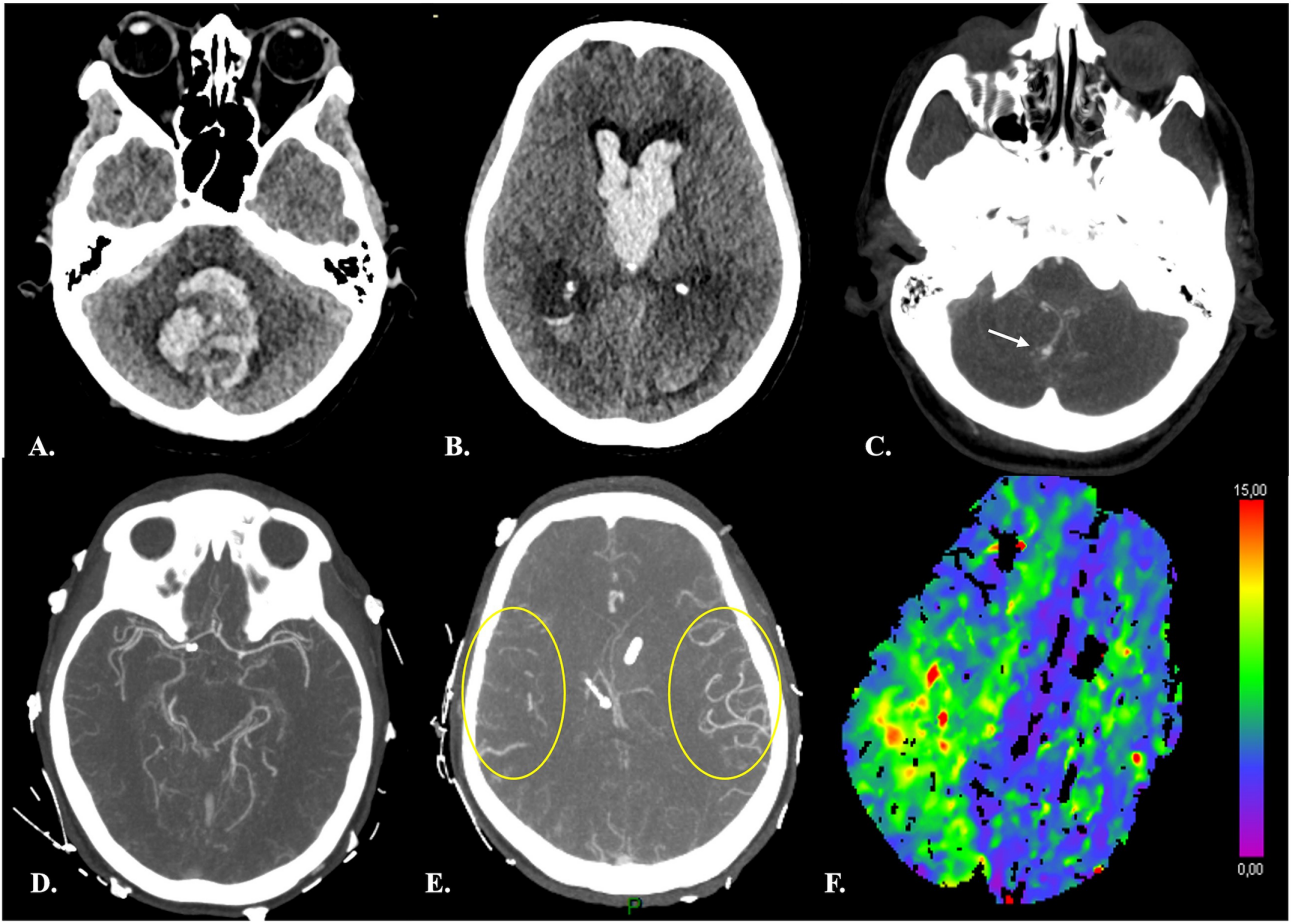
Distal vasospasm and corresponding hypoperfusion in a 42-yr-old female with Fisher 4 aSAH. **(A–B)** Brain NECT at admission showed a large hematoma centered on the fourth ventricle, intraventricular hemorrhage and hydrocephalus. CTA **(C)** demonstrated a ruptured right PICA aneurysm (*arrow*) and an unruptured right carotid-ophtalmic aneurysm (not shown). **(D–E)** At day 7 after aSAH and endovascular treatment of both aneurysms, CTA disclosed vasospasm of distal right MCA branches (*yellow circles in E, for comparison with contralateral MCA territory*). **(F)** CTP revealed prolonged TTD consistent with hypoperfusion in the corresponding ipsilateral sylvian territory.

As for AIS, in aSAH CTP is part of a multimodal CT assessment that also includes a non-enhanced CT (NECT) of the brain and a head and neck CTA (Figure 4). One important difference, however, is that detection of neurological impairment by clinical examination may not be possible in unconscious poor-grade SAH patients, which can result in an underdiagnosis of hypoperfusion and/or DCI potentially worsening outcome (58). As such, additional diagnostic methods have been implemented in clinical practice to enable earlier and more accurate diagnosis and timely treatment of impending DCI. In such clinical scenario, CTP is performed as a complement to serial transcranial color doppler (TCD) monitoring and multimodal neuro-monitoring (MMM)[see, e.g. (59)].

In line with the more complex pathogenesis of DCI as compared to AIS, the contribution of CTP in aSAH patients is also less clearly defined. It has been postulated that early detection of perfusion abnormalities irrespective of the presence of large-vessel vasospasm could help identify patients at risk before DCI symptoms or before evidence of cerebral infarctions on imaging studies [e.g. (60)]. To date, the studies that have investigated the contribution of CTP in this specific patients’ population have yielded conflicting results and there are currently no definite quantitative threshold values for defining TAR in aSAH patients (61, 62).

The main differences between CTP imaging in AIS and aSAH are summarized in Table 1.

**Table 1:** Ct perfusion (CTP) imaging in acute ischemic stroke (AIS) and aneurysmal subarachnoid hemorrhage (aSAH).

Clinical condition	Acute ischemic stroke (AIS)	Aneurysmal subarachnoid hemorrhage (aSAH)
Patients’ characteristics	Patients presenting with new-onset focal neurological deficit.	Unconscious patients in whom neurological examination is limited. CTP imaging may be performed based uniquely on abnormal TCD or MMM findings.
Aim of CTP	Providing volume estimates of the ischemic core (NVT) and ischemic penumbra (TAR)	Identifying areas of hypoperfusion (TAR) before ischemic necrosis occurs.
Common findings on CTP	One area of ischemic necrosis (i.e., the ischemic core) which is irreversibly damaged and is therefore referred to as NVT is surrounded by the ischemic penumbra, that is at risk of infarction (TAR) but potentially salvageable if recanalization is achieved. According to the concentric four-compartment brain ischemia model (63), an area of mild, “benign” hypoperfusion surrounds the ischemic penumbra, and is referred to as oligoemia. The fourth compartment is represented by the unaffected brain parenchyma. A typical case of AIS is shown in Figure 1.	One or more than one area of hypoperfusion (TAR) and/or oligoemia may be seen affecting different arterial territories (Figure 5).
Recommended method of analysis of CTP data	Automated volume measurements of NVT and TAR based on voxelwise analyses and calculation of mismatch ratios.	• Qualitative assessment of color-coded CTP maps. • ROI-based analyses.
CTP thresholds for TAR and NVT	rCBF < 30% for NVT and TMax > 6 s for TAR.	No validated threshold.
Commonly associated findings on brain NECT that may limit CTP accuracy	None.	• Intraparenchymal hemorrhage. • Intraventricular hemorrhage. • Hydrocephalus. • Intracranial devices (ventricular drain and brain oxygen and intracranial pressure monitoring systems).
Commonly associated findings on CTA	Thrombo-embolic occlusion of one arterial segment.	• Vasospasm of one or more arterial segments. • No obvious vasospasm.
CTP-based subsequent treatment decision	Endovascular thrombectomy.	Intra-arterial injection of vasodilators/angioplasty.

The table summarizes the main differences between these two clinical conditions.

TCD, transcranial color doppler; MMM, multimodal neuromonitoring; NVT, non-viable tissue; TAR, tissue at risk; rCBF, relative cerebral blood flow; TMax, time to maximum; NA, not applicable.

##### 2.4.3.1 Role of CT perfusion (CTP) in prediction of delayed cerebral ischemia (DCI)

###### 2.4.3.1.1 “Early” and “late” CT Perfusion (CTP) imaging

There is a large variability in the reported accuracy of CTP in diagnosis and prediction of DCI among authors. One important factor that influences results is the time interval of CTP from aSAH. There is some inconsistency in the literature in the definition of “early” CTP imaging, varying between ≤6 h to ≤72 h after hemorrhage. However, there seems to be a consensus on the definition of “late” CTP imaging, which refers to scans obtained >72 h after aSAH, a phase when patients are known to be more susceptible to vasospasm and DCI (64, 65). Distinguishing between early and late CTP scans may thus help reduce variability.

In a retrospective single ROI-based analysis of CTP scans obtained between day 0 and day 3 after aSAH in 75 patients, Sanelli et al. (66) observed a statistically significant reduction of CBF and prolongation of MTT in patients who subsequently developed angiographic vasospasm. The authors reported mean CBF and MTT values of 31.90 ml/100 g/min and 7.12 s respectively vs. 39.88 ml/100 g/min (*P* < 0.05) and 5.03 s (*P* < 0.01). Dong et al. (67) prospectively evaluated 191 consecutive patients with aSAH and reported that patients who developed DCI displayed lower CBF and CBV and longer MTT, TTD, TTS, and TMax in CTP scans obtained within 24 h after aSAH as compared to patients who did not develop DCI. The mean TMax had the largest AUC of 0.726, and the cutoff value of 2.240 s provided sensitivity of 73.7% and specificity of 71.6% for early prediction of DCI. Similarly, in the retrospective cohort study by Starnoni et al. (68) which included 38 aSAH patients, DCI was correlated with lower mean early CBF values (55.4 ml/100 g/min vs. 63.9 ml/100 g/min in non-DCI patients) and vasospasm with lower mean CBF and MTT values. No significant correlation, however, was found between MTT and CBV and the later occurrence of DCI. More recently, Tanabe et al. (69) reported that TMax > 4 s within 72 h from hemorrhage was an independent predictor of both “unfavorable” and “catastrophic” outcome and occurrence of DCI in a retrospective cohort of 57 aSAH patients.

Other studies, in contrast, reported no significant association between early CTP findings and the subsequent development of DCI. As reported in the meta-analysis by Cremers et al. (61), CBF, CBV, MTT, and TTP on admission did not differ significantly between patients who later developed DCI and those who did not develop DCI in a number of works (70–72). More recently, similar results were reported by Takahashi et al. (73): the authors retrospectively analyzed the clinical and imaging findings of 86 aSAH patients and observed no significant differences in the average MTT within 24 h after aSAH between territories with and without DCI, or between patients with and without DCI. Similarly, Vulcu et al. (74) did not find any significant association between early (within 24 h from aSAH) CTP findings and the subsequent development of DCI in a retrospective study including 33 patients with aSAH-related neurological deterioration. The authors reported conversely a significant association with later (days 5–14. after aSAH) CTP scans, that were obtained based on acute clinical deterioration.

A few authors, lastly, reported no significant association between quantitative CTP values and DCI but observed significant results when analyzing a combination of parameters or variations of CTP parameters. Etminan et al. (75) investigated the relation of early (<12 h from aSAH) CTP and clot volume with DCI and clinical outcome and reported that odds for poor outcome were significantly higher in case of concomitant increase of early MTT and clot volumes, as opposed to exclusive early MTT or clot volume increase. In Rodriguez-Régent et al. (76), MTT and CBF values were compared between patients with DCI and patients without DCI for previously published optimal cutoff values and for variations of MTT (ΔMTT) and of CBF (ΔCBF) values between day 0 and day 4 after aSAH. The authors concluded that published optimal cutoff values did not predict DCI, either at day 0 or at day 4. Conversely, ΔMTT and ΔCBF significantly differed between patients with and without DCI, with a CBF decrease of 7.6 ml/min/100 g and an MTT increase of 0.91 s being accurate predictors of DCI.

More consistent results are found when using CTP imaging during a later time-window, from 3 to 14 days after aSAH. This time-period is indeed known to be associated with a higher incidence of vasospasm and DCI (64, 65). Wintermark et al. (26) retrospectively evaluated 35 NECT-CTA-CTP, TCD, and DSA examinations of 27 aSAH patients performed because of a clinical suspicion of vasospasm after a median delay of 7 days after hemorrhage (range, 5–14 days), and reported that MTT with a threshold of 6.4 s was the most accurate parameter for the diagnosis of angiographic vasospasm, with a very high NPV of 98.7%.

In the past two decades, along with the greater insight into the pathophysiology of aSAH complications, most studies focused on the association between CTP and DCI rather than vasospasm. In a retrospective study including 96 patients with aSAH, Sanelli et al. (77) reported that CTP between days 6 and 8 after aSAH yielded a sensitivity of 78% and a specificity of 66% for the development of permanent neurological deficits and a sensitivity of 88% and a specificity of 59% for the development of DCI. Kunze et al. (78) retrospectively evaluated 53 patients with aSAH who underwent CTP between days 3 and 10 after aSAH and additionally at any other time point when cerebral vasospasm was suspected, and reported that CTP had higher accuracy, sensitivity and negative predictive value than TCD for predicting vasospasm, with TTP being the most accurate parameter (sensitivity 0.93). Killeen et al. (79) reported sensitivity and specificity values of 84% and 73% respectively for the detection of infarction and/or permanent neurological deficit in a retrospective ROI-based study of 97 consecutive aSAH patients between day 6 and day 8 after aSAH. Shi et al. (80) analyzed quantitative and semi-quantitative CTP parameters (CBV, CBF, MTT, TTP, rCBV, rCBF, and MTT and TTP difference (ΔMTT, ΔTTP) were compared between the DCI and non-DCI (NDCI) groups at three time points: whereas only a weak correlation was observed with baseline CT, more significant results were observed with CTP imaging performed on days 4 and 7 after aSAH. Similar conclusions were drawn in the previously cited work by Vulcu et al. (74), who reported that MTT, TTP, and TTD were significantly prolonged and CBF decreased in patients with DCI compared with the not symptomatic hemisphere and with the baseline CTP.

Lastly, Hofmann et al. (81), similar to Rodriguez-Régent et al. (76), analyzed the heterogeneity of MTT rather than absolute values, using the coefficient of variation of MTT in late CTP imaging, and reported that this parameter correlates significantly not only with the initial World Federation of Neurosurgical Societies (WFNS) or Fisher grade, but also, and most importantly, with a worse neurological outcome after 6 months. The heterogeneity of MTT is here considered an indicator of the capillary transit time heterogeneity (CTH), i.e., the microvascular blood distribution across capillaries, which is known to decrease the oxygen supply to cerebral tissue thereby causing hypoxia. MTT heterogeneity has thus been reported to possibly play a crucial role in causing DCI (82, 83).

The results of the above-mentioned studies are summarized in Tables 2, 3.

**Table 2:** Role of “early” CT perfusion (CTP) in prediction of delayed cerebral ischemia (DCI).

Author(s) (year)	Time interval of CTP imaging with respect to aSAH	Population (*n*)	Study design	Method of analysis	CTP parameter(s) that were analyzed	Correlation between CTP and DCI (Y/N)	Most accurate CTP parameter or CTP finding predictive of DCI
Dong et al. (67)	<24 h	191	Prospective	ROI-based. 32 ROIs were hand-drawn on five slices in the cortical flow territories of the ACA, MCA, PCA, basal ganglia, and cerebellum.	Mean values (the values of 32 ROIs for each parameter were averaged in each patient) and lowest values (i.e., the minimum values of CBF and CBV, and the maximum values of MTT, TTD, TTS, and TMax of single ROIs for each patient)	Y	Mean TMax
Duan et al. (84)	<72 h	54	Prospective	ROI-based. 8 ROIs were manually drawn in the ACA, MCA, and PCA territories, basal ganglia, anterior and posterior watershed zones.	rCBV, rCBF, rMTT	Y	rCBF
Lagares et al. (60)	<24 h	39	Prospective	ROI-based. ROIs were drawn in the white matter of the major arterial territories and in the basal ganglia of both cerebral hemispheres.	Individual ROI and averaged (m) values of MTT, TTP, CBV and CBF	Y	mMTT ≥5.9s
Lanterna et al. (70)	<72 h	41	Prospective	Qualitative assessment of CTP color-coded maps.	Topography of hypoperfusion	Y	Hypoperfusion of watershed zones.
Rijsdijk et al. (71)	<72 h	27	Prospective	ROI-based. 8 Eight ROIs were drawn in the cortical gray matter or basal ganglia bilaterally in the peripheral and deep flow territories ACA and MCA.	CBF	N	NA
Rodriguez-Régent et al. (76)	Day 4	47	Prospective	ROI-based. 28 contiguous ROIs were manually drawn on 4 sections in the ACA, MCA, PCA territories and basal ganglia.	Variations of MTT (ΔMTT) and of CBF (ΔCBF) values	Y	ΔMTT of 0.9 s and ΔCBF of −7.6 ml/100 g/min
Sanelli et al. (66)	<72 h	75	Retrospective	ROI-based. 22 ROIs were drawn in the ACA, MCA, PCA territories and basal ganglia.	Mean CBF, CBV and MTT	Y	CBF and MTT
Shi et al. (80)	<24 h	39	Prospective	ROI-based. A total of 16 ROIs were manually drawn on three transverse sections in the ACA and MCA territories and thalamic and caudate nuclei.	CBV, CBF, MTT, TTP, rCBV, rCBF, and MTT and TTP difference (ΔMTT, ΔTTP)	N	NA
Starnoni et al. (68)	<48 h	38	Retrospective	ROI-based. 4 ROIs were manually drawn in the subcortical white matter of the ACA and MCA territories.	CBF, CBV and MTT	Y	Mean CBF
Takahashi et al. (73)	<24 h	86	Retrospective	26 ROIs in the ACA, MCA, PCA territories and basal ganglia	average MTT (aMTT), i.e., average of all measured values of the parameter in the different ROIs	N	NA
Tanabe et al. (69)	<72 h	57	Retrospective	ROI-based. 12 automatically defined ROIs for each cerebral hemisphere, on one slice at the level of the basal ganglia and one slice at the level of the body of the lateral ventricle.	TMax > 4s	Y	TMax > 4s
van der Schaaf et al. (72)	< 72 h	46	Prospective	ROI-based. 8 ROIs were drawn in the cortical territories of the ACA and MCA and in the basal ganglia.	CBV, CBF, MTT and TTP. Quantitative and semiquantitative (ratios for CBF and CBV, and differences for MTT and TTP with respect to contralateral ROIS) values.	N/Y	DCI was not significantly related with quantitative CTP values but was significantly relaed with perfusion asymmetry of CTP parameters.
Vulcu et al. (74)	<24 h	56	Retrospective	ROI-based. 10 ROIs were manually drawn in the ACA, MCA and PCA territories and in the basal ganglia.	MTT, TTP, TTD, CBF, and CBV	N	NA

The table lists the main studies that have investigated the accuracy of early” CTP parameters in predicting DCI. The definition of early varies between ≤6 h to ≤72 h after hemorrhage.

**Table 3:** Role of “late” CT perfusion (CTP) in prediction of delayed cerebral ischemia (DCI).

Author(s) (year)	Time interval of CTP imaging with respect to aSAH	Population (*n*)	Study design	Method of analysis	CTP parameter(s)	Correlation between CTP and DCI (Y/N)	Most accurate CTP parameter or CTP finding predictive of DCI
Hofmann et al. (81)	Days 3–21	132	Retrospective	ROI-based	Coefficient of variation of MTT (cvMTT)	Y	cv MTT
Killeen et al. (79)	Days 6 and 8	97	Retrospective	Qualitative assessment of CTP color-coded maps	CBF and MTT	Y	NA* * the authors report an overall sensitivity of 0.84 and specificity for the technique in detecting DCI.
Kunze et al. (78)	Days 3–10	53	Retrospective	Qualitative assessment of CTP color-coded maps	CBF, CBV, TTP	Y	TTP
Lanterna et al. (70)	Days 4–8 and 9–15	41	Prospective	Qualitative assessment of CTP color-coded maps	Topography of hypoperfusion	Y	Hypoperfusion of arterial territories (rather than watersed areas)
Pham et al. (85)	Days 3–14	38	Prospective	• ROI-based. • Qualitative assessment of CTP color-coded maps	MTT, TTP, TTD, CBF, and CBV	Y	Qualitative assessment of TTP color-coded maps (with 0.93 sensitivity and 0.67 specificity)
Sanelli et al. (77)	Days 6–8	96	Retrospective	ROI-based analysis. A total of 24 ROIs were drawn in the cerebral cortex of the ACA, MCA and PCA territories.	Mean CBF, CBV and MTT	Y	CBF < 30.5 ml/100 g/min and MTT >5.0 s
Seyour et al. (59)	When acute deterioration occurred	211	Retrospective	Qualitative assessment of CTP color-coded maps	TMax > 4s and TMax > 6s	Y	TMax >4 s
Shi et al. (80)	Days 4 and 7	39	Prospective	ROI-based. A total of 16 ROIs were manually drawn on three transverse sections in the ACA and MCA territories and thalamic and caudate nuclei.	rCBV, rCBF, ΔMTT and ΔTTP that were calculated using mirror ROIs	Y	CBF, rCBF, and ΔMTT were significantly different between DCI and non-DCI groups.
Vulcu et al. (74)	Days 5–14, when acute deterioration occurred	56	Retrospective	ROI-based. 10 ROIs were manually drawn in the ACA, MCA and PCA territories and in the basal ganglia.	MTT, TTP, TTD, CBF, and CBV	Y	TTD > 4.7 s

The table lists the main studies that have investigated the accuracy of “late” CTP parameters in predicting DCI.The term “late” refers to >72 h after aSAH.

###### 2.4.3.1.2 CT Perfusion (CTP) parameters as predictors of delayed cerebral ischemia (DCI)

According to a recent meta-analysis aimed at assessing the accuracy of early CTP parameters for predicting DCI, including fifteen studies with 882 aSAH patients, the most accurate predictor was MTT (62). A similar conclusion was drawn in studies that were not included in the meta-analysis by Han et al. (62). Etminan et al. (75), for instance, reported that MTT measured within 12 h from hemorrhage was able to detect patients at a higher risk of DCI and poor neurological outcome at six months. In the previously cited work by Wintermark et al. (26), MTT considered alone represented the most sensitive parameter for diagnosing vasospasm. The authors also report that TTP was not discriminative, and highlight that, as opposed to MTT, TTP values are calculated without any AIF, which renders TTP very sensitive to extracerebral or precerebral variables, such as cardiac output, extra- or intracranial arteries stenosis, or the intravenous injection of the iodinated contrast material bolus. MTT was also significantly correlated with arterial diameter and neurological deficits in Laslo et al. (86). Similarly, in a previous animal model, MTT increase 1 h after experimental SAH in rabbits independently predicted mortality within 48 h of SAH and MTT on day 2 predicted development of moderate- to severe delayed vasospasm. Han et al. (62), nonetheless, in line with the previous meta-analysis by Cremers et al. (61), observed that the CTP thresholds employed in the studies they analyzed were incomparable. Also, a number of studies have considered variations and heterogeneity of MTT as predictors of DCI rather than MTT alone (76, 81).

In contrast with these observations, Takahashi et al. (73) reported that average MTT did not show any significant correlation with the subsequent development of DCI. Other works in the literature showed that other CTP parameters than MTT were most significantly associated with DCI. Pham et al. (85) prospectively evaluated 38 patients during two weeks after aSAH and observed that qualitative assessment of TTP color-coded maps performed best for prediction of secondary infarction with 93% sensitivity and 67% specificity. The CTP parameter with the highest sensitivity (93%) for the detection of angiographic vasospasm, although with a low specificity (27%), was TTP also in the study by Kunze et al. (78). In Dong et al. (67), the most accurate parameter for early prediction of DCI was the mean TMax. In agreement with Dong et al. (67), Tanabe et al. (69) in a retrospective study on 57 aSAH patients reported that cortical cerebral flow insufficiency, defined as TMax > 4 s—within 72 h from hemorrhage—was an independent predictor of both “unfavorable” and “catastrophic” outcome and DCI occurrence. More recently, our group used prolongation of TMax > 4 s to define hypoperfusion regardless of the presence of cerebral vasospasm and in the absence of intracranial hypertension. We observed that detection and treatment of cerebral hypoperfusion on CTP scans performed because of altered MMM or clinical findings that did not fulfill standard DCI criteria resulted in a significant reduction in the number of DCI- related infarctions (59).

###### 2.4.3.1.3 Quantitative and semi-quantitative measurements of CT perfusion (CTP)

Although some methodological differences exist in the literature, measurements of CTP parameters in research studies are mostly based on regions of interest (ROIs) (60, 67–69, 71, 72, 74, 76, 80, 84, 87–91). Semi-quantitative measurements are frequently used, to avoid confounding factors related to inter-individual variation, physiological conditions, and observer-dependent postprocessing differences (61).

Some differences among authors concerning the location of the ROIs are likely related to advances in CTP imaging during the past two decades. One important drawback of early CTP imaging was the limited anatomical coverage, whereas modern CT scanners allow for whole-brain coverage. It is therefore not surprising that in the earliest CTP studies ROIs were drawn on only one or two slices typically at the level of the basal ganglia [e.g.,: 2008 (25, 60, 71, 77, 87)]. More recent studies present nonetheless some differences concerning ROIs location. In Sun et al. (91), perfusion was measured in ROIs that were drawn in the basal ganglia and in the cortex of the ACA and MCA territories bilaterally. The PCA territory was excluded from analyses due to the reported incidence of partial volume effects of large veins in the posterior fossa with consequent high variability in quantitative values (87, 92). The PCA territory was also excluded by Shi et al. (80). The PCA territory was conversely included in Duong et al. (67), who drew ROIs on the maximum-intensity-projection map of five slices in the cortical flow territories of the ACA, MCA, PCA, basal ganglia, and cerebellum. In Starnoni et al. (68), ROIs were manually drawn in the subcortical white matter of both ACA and MCA rather than in cortical territories. In Malinova et al. (65), whole-vessel territories of ACA, MCA, and PCA were drawn and included in the VOIs. Hofmann et al. (81), conversely, used automated definition of ROIs in the anterior, middle, and posterior cerebral artery territories as well as the anterior and posterior borderzones.

The existing literature differs also concerning semi-quantitative measurements. These can be obtained by using interhemispheric (lowest to highest) ratios (87), relative CTP values obtained by comparing values of the most affected cerebral hemisphere with the less affected cerebral hemisphere (84), ratios for corresponding ROIs of the two cerebral hemispheres (71, 80, 91), or absolute variations of CTP parameters (76).

Tables 2, 3 also detail the method of analysis of CTP data and indicate the CTP parameter that best predicted DCI in each study.

##### 2.4.3.2 Role of CT perfusion (CTP) in the assessment of response to treatment

**Figure 5:**
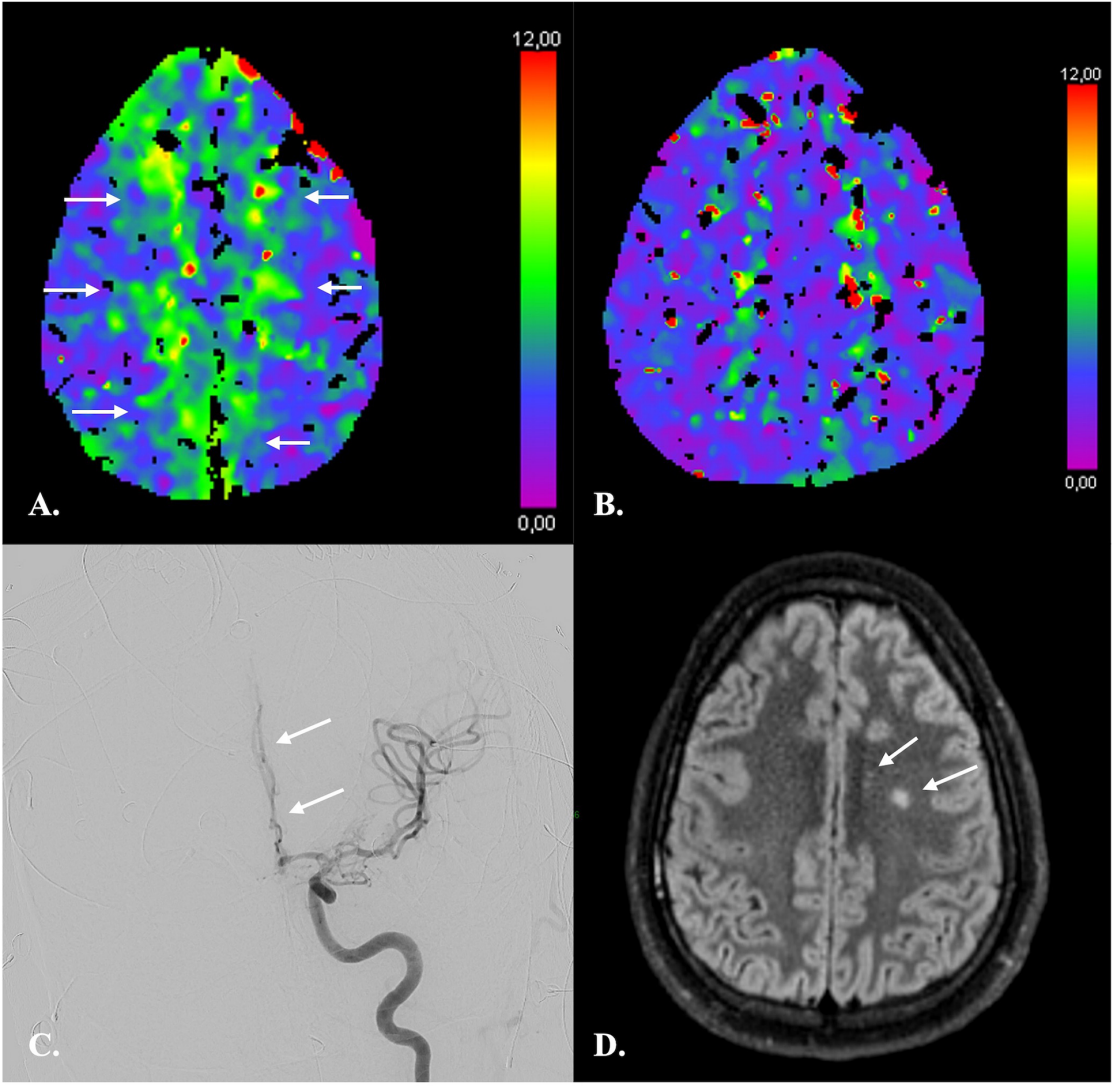
CTP imaging in the response assessment to ERT in a 26-year-old male with fisher 4 aSAH. **(A)** At day 11 after aSAH, CTP revealed a moderately increased TMax consistent with hypoperfusion of both ACA territories (*arrows*). **(B)** CTP following ERT on day 12 demonstrated almost complete regression of hypoperfusion. No new areas of hypoperfusion appeared. **(C)** DSA confirmed vasospasm of multiple intracranial arteries and in particular of the A2 segment of the left anterior cerebral artery (*arrow*). ERT consisted in intra-arterial injections of nimodipine and selective angioplasty of supraclinoid internal carotid arteries and M1 segment of the left middle cerebral artery as in Guenego et al. (96). **(D)** On MRI one month after aSAH, only two small infarcts of the left centrum semi-ovale were demonstrated.

The past two decades have witnessed important technical advances not only in CTP imaging but also in ERT methods. Despite the considerable heterogeneity among the neurointerventional community regarding vasospasm endovascular management (93–95), CTP imaging has become part of the multimodal assessment of patients who undergo ERT. CTP is performed serially with the aims of assessing local response to treatment and to rule out the appearance of new areas of hypoperfusion that would warrant further treatment (Figure 5). To date, only a few studies have investigated such effects. Steiger et al. (39) investigated the effects ERT on CTP parameters in 98 patients who suffered from DCI, by comparing the T4 volume (i.e., TTP delay of more than 4 s) and the TTP delay of the ischemic core compared to a reference ROI before and after treatment with milrinone and norepinephrine–based hyperdynamic therapy. They observed that patients suffering of secondary neurological decline had T4 volumes of 40 cc and an average focal TTP delay of 2.5 s, and that following ERT median T4 volume was reduced to 10 cc and average focal TTP delay to 1.7 s. More recently, Guenego et al. (96) investigated the effects of mechanical angioplasty of 48 arterial segments in 8 patients with medically refractory cerebral vasospasm and reported a significant decrease of brain hypoperfusion (81% of the mean TMax as measured in pre-defined ROIs in the ACA and MCA territories) along with an increase of vessel diameter (+81%).

#### 2.4.4 Limitations of CT perfusion (CTP) imaging

CTP has several limitations that need to be considered when interpreting CTP results.

**Figure 6:**
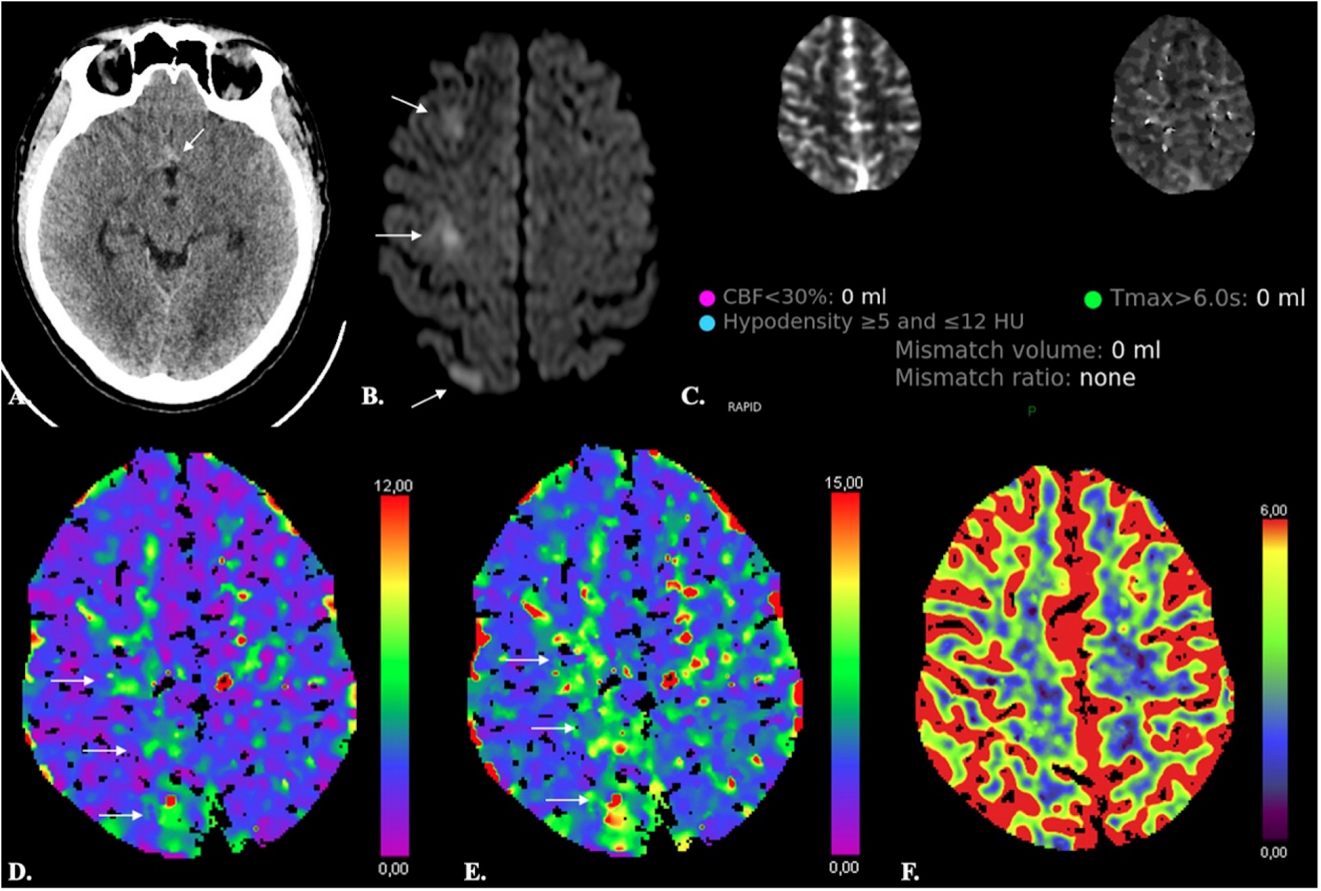
Limited sensitivity of CTP in detecting small ischemic lesions. A 38-year-old male with Fisher 1 aSAH (*arrow in A*) due to rupture of an ACom aneurysm treated with endovascular coiling developed abrupt onset paresis of the left inferior limb on day 8 after aSAH. Automated results of both the RAPID (**C**) and SyngoVia (*not shown*) CTP software did not reveal any significant decrease of CBF or CBV (**F**) and no significant increase of Tmax. On inspection, however, Tmax and TTD were moderately increased (*arrows in D and E*). Diffusion-weighted MRI (**A**) revealed three foci of acute ischemia in the right ACA territory (*arrows in B*).

One drawback of CTP imaging is its relatively limited spatial resolution, and consequently a limited sensitivity in detecting lacunar or small subcortical infarcts. In one study, CTP had a sensitivity of only 62% in comparison to diffusion-weighted MRI. The technique, however, was more sensitive than NECT, which displayed even lower (i.e., 19%) sensitivity values (97). In addition, although small infarcts are usually not detected by conventional CTP thresholds, visual inspection of in particular MTT, TTP, TMax and TTD maps has been shown to yield moderate sensitivity and high specificity for detection of these lesions (Figure 6) (97–99). Sensitivity for infratentorial lesions is, however, low (97).

**Figure 7:**
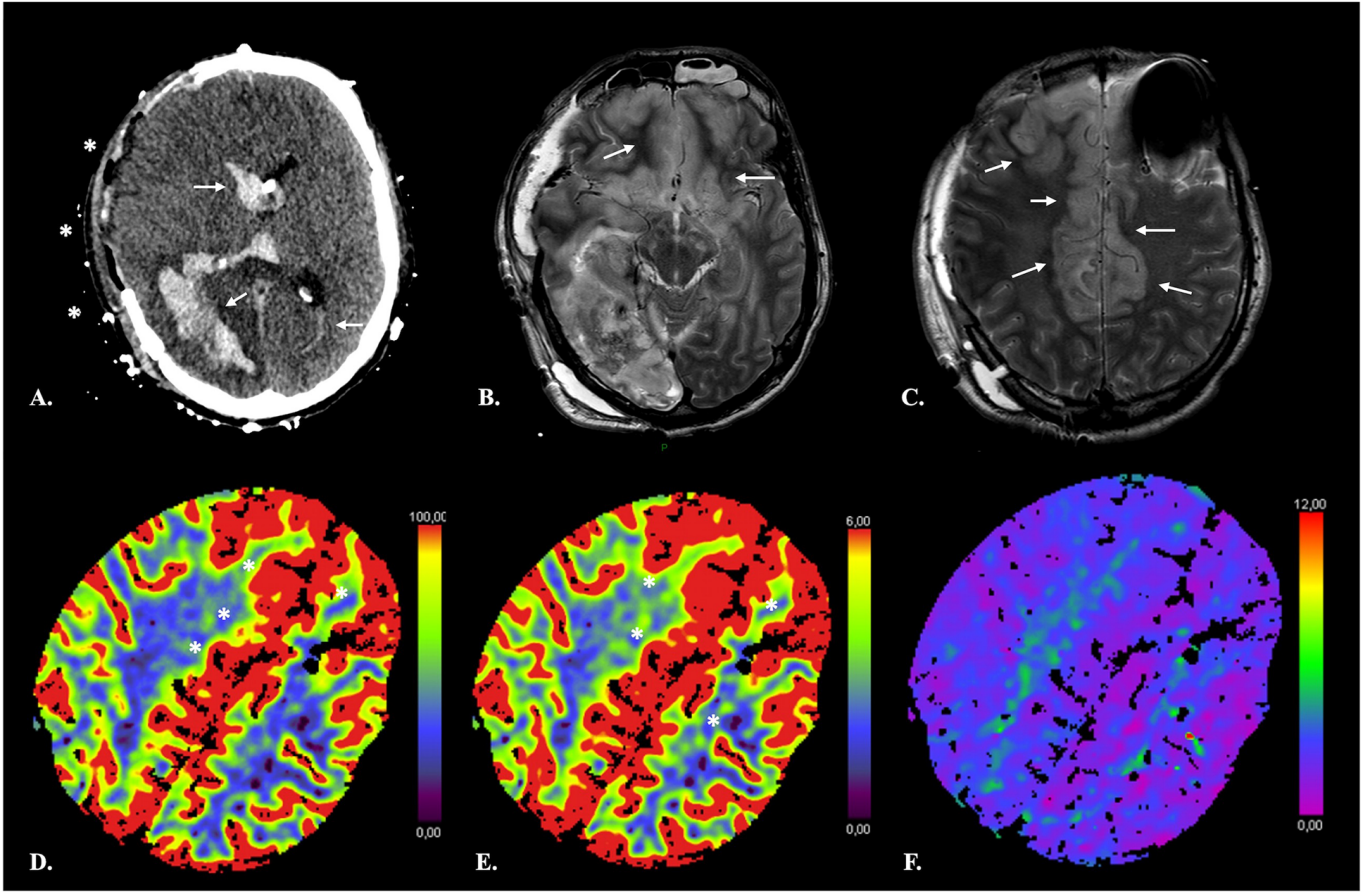
False-negative CTP results in a 41-year-old male with extensive ischemia of the anterior cerebral cortex due to subfalcine herniation following rupture of a right parieto-occipital AVM. NECT of the brain **(A)** shows a subacute right parieto-occipital hematoma with associated intraventricular hemorrhage (*arrows*). Midline shift has regressed following right hemispheric craniectomy (*). Axial T2-weighted TSE images **(B,C)** reveal swelling and increased signal intensity of the cortex in the ACA territory bilaterally consistent with ischemia. On CTP, the infarcted cortex displays increased CBF **(D)** and CBV **(E)** with no obvious TMax **(F)** abnormality resulting in negative computation of both NVT and TAR by the software.

Another limitation of using CTP thresholds is the inability to detect NVT after recanalization or even in patients with persistent arterial occlusion who present a late increase in blood flow in irreversibly damaged tissue due to improved perfusion via collateral blood vessels (100, 101). As highlighted by Demeestere et al. (102), the infarcted tissue will not be identified as NVT on CTP if CBF exceeds the threshold for ischemic core detection. Thorough inspection of NECT images for subacute or established infarction is thus mandatory for CTP interpretation (Figure 7). This is especially true in aSAH patients with DCI undergoing serial ERT and serial imaging.

**Figure 8:**
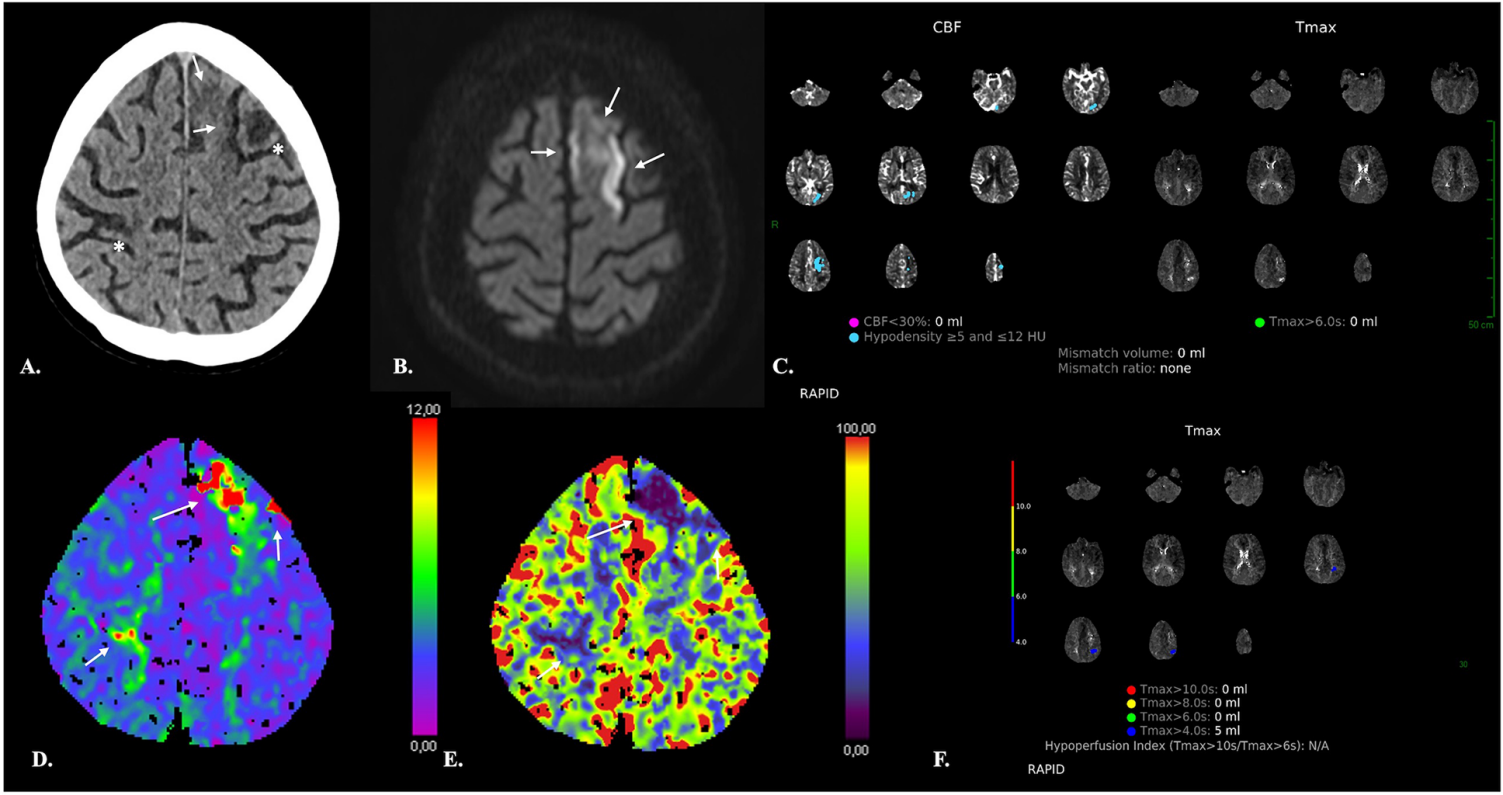
Confusing CTP features of acute superimposed on late-stage ischemic changes in an 89-year-old male with abrupt onset of left upper and lower limb paresis. **(A)** NECT of the brain demonstrates right rolandic and left middle frontal cortical hypodensities consistent with late-stage ischemic changes (*) and ill-defined, moderate hypodensity of the cortex and subcortical white matter of left superior frontal gyrus (*arrows*). **(B)** MRI performed 4 days after the CT scan demonstrates restricted diffusion consistent with acute ischemia (*arrows*). **(C,F)** The RAPID software discloses no corresponding NVT or TAR but demonstrates moderate left parietal hypoperfusion which is conversely MRI-negative. **(D,E)** Qualitative assessment of the SyngoVia CTP maps reveals changes in the Tmax and CBF in both recent and chronic lesions (*arrows*), which may be therefore difficult to distinguish on the basis of CTP only.

Late-stage infarcts, while frequently obvious on NECT, may also display confusing CTP features (103). Results of automated CTP measurements may not allow to distinguish late-stage from relatively recent infarcts, nor may qualitative assessment of color-coded maps, again rendering correlation with NECT images essential to avoid misinterpretation (Figure 8).

One important technical pitfall may occur in the presence of severe stenosis of large arteries or other conditions such as low cardiac output or cardiac arrhythmia which are responsible for a reduced upstream arterial flow (104). A delay in the arrival of the infused bolus of contrast material results in truncation of TACs, i.e., ending earlier than appropriate TACs. This occurs because of the relatively short imaging time of CTP and precludes calculation of diagnostic CTP maps (104).

Stenosis of intracranial arteries may also lead to confusing CTP results by causing hypoperfusion that may be misclassified as ischemic penumbra in the acute setting (105, 106). The most consistent and reproducible CTP finding is MTT prolongation, while CBF and CBV maps show variable changes (106, 107). These pitfalls stress the importance of thoroughly assessing not only NECT of the brain but also concurrent head and neck CTA to evaluate for regions of arterial stenosis.

The technique is sensitive to beam-hardening artefacts, which may be especially pronounced in aSAH patients with treated aneurysms and intracranial devices. Although easy to recognize on NECT, artefacts in the skull base or orbits represent a potential source of overestimation of penumbral volume (55, 104).

Iterative metal artifact reduction algorithms (iMAR, Siemens Healthcare) have been introduced during the past decade and have shown to significantly reduce metal artefacts in different body parts (see, e.g., (108, 109), and most importantly in brain NECT and CTA after coiling or clipping [see, e.g., (110–112),]. More recently, the iMAR algorithm has been applied also to CTP, with favorable results (113). Hakim et al. (113) compared the CTP maps generated from 58 datasets both with and without iMAR in 32 patients with aSAH, and showed that application of iMAR reduced artifacts and significantly improved image quality. The authors, however, observed that the performance of iMAR may differ depending on the type and size of metal and recommend iMAR application only in patients with strong metal artifacts. It is interesting to note that an increase of metal artefacts, with no improvement of image quality after application of the iMAR algorithm, has been observed on both source images and color-coded CTP summary maps in association with the use of orbit shields, which are therefore not recommended in CTP (114).

**Figure 9:**
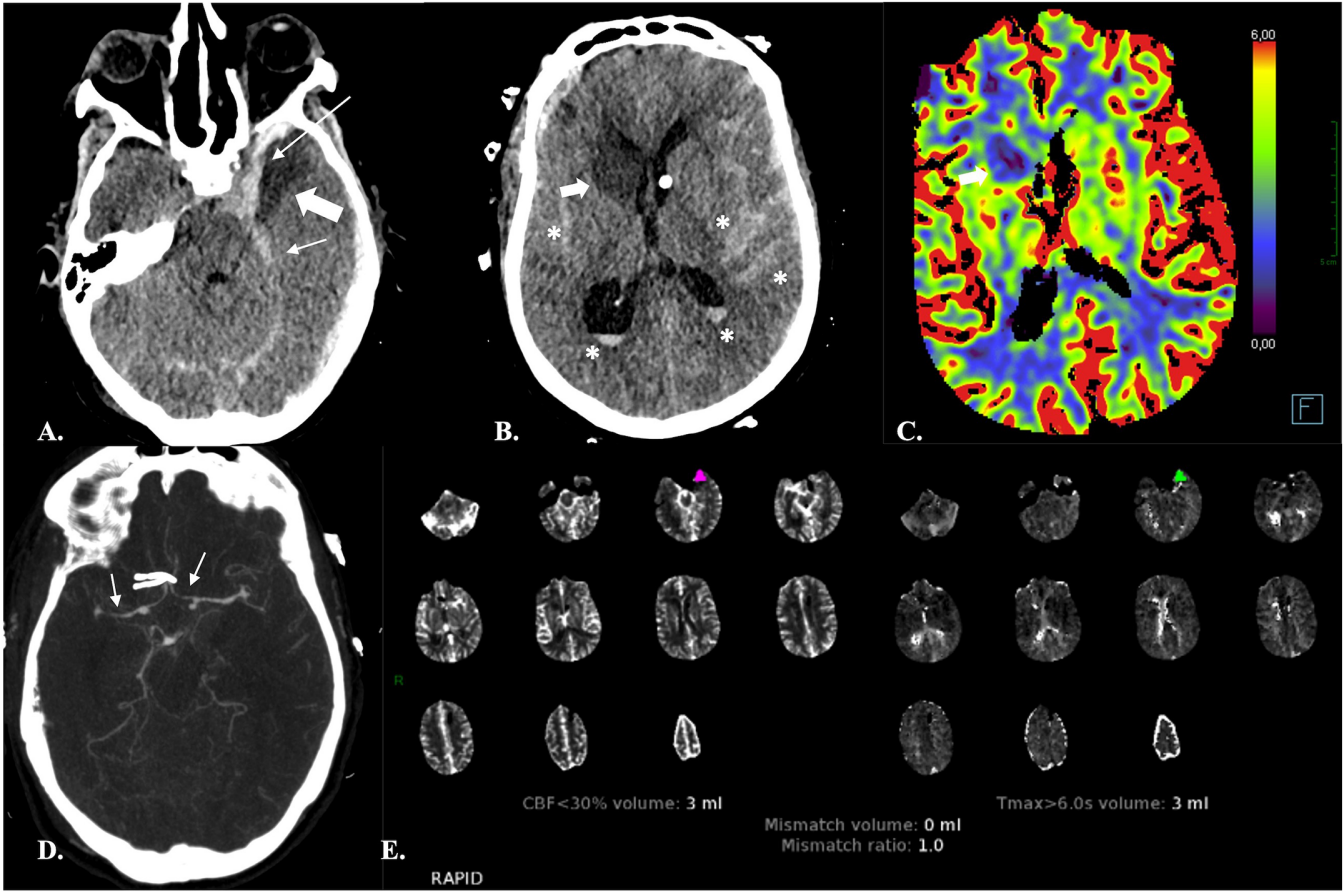
Discordance between software. 66-year-old female with Fisher IV aSAH (* in A and B) and associated subdural hematomas (*arrows in A*), who underwent surgical treatment of an ACom aneurysm and endovascular repair of a left PCom aneurysm. NECT of the head **(A,B)** on day 7 after aSAH revealed ischemic lesions of the head of the right caudate nucleus and left temporal pole (*bold arrows*). The left temporal lesion was correctly identified by both the RAPID and SyngoVia software. The RAPID software, however, failed to identify the right caudate nucleus lesion. The latter appeared as an area of decreased CBF and CBV on the SyngoVia CT perfusion maps (*bold arrow in C*). CTA revealed narrowing of the A1-A2 and M1 segments bilaterally consistent with vasospasm (*arrows in D*).

Finally, it seems important to mention that a potential limitation is represented by discordance between commercially available software. Multiple software products that produce perfusion maps and estimate volumes of NVT and TAR are available. However, substantial differences exist between vendors (115, 116), that may influence patient selection for reperfusion therapy in the setting of AIS triage and may be responsible for bias and confounding in longitudinal follow-up of aSAH patients (Figure 9). Kudo et al. (115) reported an important variability in both the qualitative and quantitative results of CTP in AIS patients by using different software, even when using identical source data, which the authors attributed to differences in tracer-delay sensitivity.

The CTP limitations discussed here above are summarized in Table 4.

**Table 4:** Limitations of CT perfusion imaging.

Limitations of CTP
• Limited sensitivity for small infarcts
• Inability to detect NVT after recanalization or collateral flow
• Inability to distinguish late-stage from recent infarcts
• Impaired calculation of CTP maps in the presence of a reduced upstream arterial flow (e.g., arterial stenosis, low cardiac output, cardiac arrhythmia)
• Sensitivity to beam-hardening artefacts
• Discordance between commercially available software

### 2.5 Additional considerations and conclusions

Despite the heterogenous results concerning the contribution of CTP in aSAH, and despite the lack of a standardized method of data analysis, the use of CTP imaging has been growing in this patients’ population during the past two decades. As highlighted by Sanelli et al. (77), there are several advantages in using CTP in this critically ill population, despite its limitations. First, the technique is widely available. Second, it has relatively short acquisition times, especially as compared to MRI, which is of the utmost importance in unstable ICU patients (117). High-field MRI has additional limitations, the main ones being susceptibility to ferromagnetic material or contraindications due to the equipment needed by an ICU patient during image acquisition (117). Also, it allows one-stop NECT, CTA, and CTP acquisition whereas MR angiography and dynamic-susceptibility-contrast (DSC) MR perfusion cannot be achieved during the same examination. Another advantage of CT in aSAH patients is that the technique is less invasive than DSA and can be performed repeatedly. CTP has also proven more accurate than cerebral ultrasound perfusion imaging (UPI), a technique that was introduced during the past two decades showing promising results (118). In a recent work on 30 aSAH patients, cerebral UPI has shown to enable detection of cerebral hypoperfusion, with the left-right difference of TTP values being the most sensitive finding (119). Concomitant assessment of cerebral vasospasm by using TCD, also, has proven to be less accurate than CTA (120, 121), thus rendering the replacement of CT by ultrasound in this patients’ population unlikely.

CTP is performed in aSAH with the aim of identifying patients at risk of DCI. While unable to reliably predict DCI if performed in an “early” (<72 h from aSAH) phase, the technique has proven more informative when performed in a later (>72 h) time-window. In addition, it seems important to highlight the crucial role of CTP on admission as a baseline reference for subsequent CTP studies.

Very importantly, the technique has proven an informative tool in the assessment of the response to ERT, with cerebral perfusion in the setting of secondary cerebral ischemia following aSAH being measurably improved by ERT (39, 96).

Many authors advocate for standardization of methods of analyzing CTP and for defining CTP thresholds for both NVT and TAR in aSAH. While validated in AIS, automated measurements from voxelwise analyses seem to present major limitations in aSAH due to the high incidence of DCI-unrelated parenchymal damage in this patients’ population. Intraparenchymal hemorrhage, hydrocephalus or intracranial devices may indeed be responsible for severe artefacts that render automated measurements unreliable.

In assessment of response to ERT, an additional limitation of voxelwise analyses is the dynamic nature of vasospasm, which may regress in one territory following selective treatment on one CTP scan while at the same time affecting a new arterial territory, with unchanged values of the total volume of brain parenchyma considered at risk (TAR) on subsequent CT scans. ROI-based analyses seem thus more appropriate in this specific clinical setting, allowing to specifically evaluate the CTP changes in a given arterial territory while excluding areas of damaged brain tissue that would result in false-positive CTP findings.

While most research studies are indeed ROI-based, qualitative assessment of color-coded maps seems a valid alternative in clinical practice but requires thorough inspection of corresponding NECT and CTA images to avoid misinterpretation. In addition, lower TAR thresholds (i.e., TMax > 4 s) seem more appropriate in aSAH as compared to AIS. AIS thresholds underestimate both the infarct core and hypoperfusion, with the aim of favouring patients’ selection for thrombectomy. CTP in aSAH, differently, needs to be as sensitive as possible in the detection of TAR.

Given the discordance between commercially available software and differences in methodological analyses, standardization seems difficult to achieve but consistency is mandatory across aSAH patients in each single institution, allowing for accurate follow-up and between-patient comparison.

Despite these complexities, the use of CTP imaging in routine aSAH care as a complement to TCD and MMM seems indispensable, in that it is easy and rapid to perform and allows individualized treatment in particular of unconscious patients based not only on vascular assessment but most importantly on brain tissue functional status.

## References

[1] Chung CY Hu R Peterson RY Allen JW. Automated processing of head CT perfusion imaging for ischemic stroke triage: a practical guide to quality assurance and interpretation. AJR. (2021) 2021(217):1401–17. 10.2214/AJR.21.26139 34259036

[2] Macdonald RL Schweizer TA. Spontaneous subarachnoid hemorrhage. Lancet. (2017) 389(10069):655–66. 10.1016/S0140-6736(16)30668-7 27637674

[3] Feigin VL Lawes CMM Bennett DA Anderson CS. Stroke epidemiology: a review of population-based studies of incidence, prevalence, and case-fatality in the late 20th century. Lancet Neurol. (2003) 2(1):43–53. 10.1016/s1474-4422(03)00266-7 12849300

[4] Etminan N Chang HS Hackenberg K de Rooij NK Vergouwen MDI Rinkel GJ Worldwide incidence of aneurysmal subarachnoid hemorrhage according to region, time period, blood pressure, and smoking prevalence in the population. A systematic review and meta-analysis. JAMA Neurol. (2019) 76(5):588–97. 10.1001/jamaneurol.2019.0006 30659573 PMC6515606

[5] Nieuwkamp DJ Setz LE Algra A Linn FH de Rooij NK Rinkel GJ. Changes in case fatality of aneurysmal subarachnoid haemorrhage over time, according to age, sex, and region: a meta-analysis. Lancet Neurol. (2009) 8(7):635–42. 10.1016/S1474-4422(09)70126-7 19501022

[6] Wahood W Rizvi AA Alexander AY Yolcu YU Lanzino G Brinjikji W Trends in admissions and outcomes for treatment of aneurysmal subarachnoid hemorrhage in the United States. Neurocrit Care. (2022) 37(1):209–18. 10.1007/s12028-022-01476-5 35304707

[7] Gouvêa Bogossian E Diaferia D Minini A Ndieugnou Djangang N Menozzi M Peluso L Time course of outcome in poor grade subarachnoid hemorrhage patients: a longitudinal retrospective study. BMC Neurol. (2021) 21:196. 10.1186/s12883-021-02229-1 33985460 PMC8117582

[8] Al-Khindi T Macdonald RL Schweizer TA. Cognitive and functional outcome after aneurysmal subarachnoid hemorrhage. Stroke. (2010) 41:519–36. 10.1161/STROKEAHA.110.581975 20595669

[9] Taufique Z May T Meyers E Falo C Mayer SA Agarwal S Predictors of poor quality of life 1 year after subarachnoid hemorrhage. Neurosurg. (2016) 78:256–64. 10.1227/NEU.0000000000001042 26421590

[10] Durrant JC Hinson HE. Rescue therapy for refractory vasospasm after subarachnoid hemorrhage. Curr Neurol Neurosci Rep. (2015) 15(2):521. 10.1007/s11910-014-0521-1 25501582 PMC4282184

[11] Vergouwen MD Vermeulen M van Gijn J Rinkel GJ Wijdicks EF Muizelaar JP Definition of delayed cerebral ischemia after aneurysmal subarachnoid hemorrhage as an outcome event in clinical trials and observational studies: proposal of a multidisciplinary research group. Stroke. (2010) 41(10):2391–5. 10.1161/STROKEAHA.110.589275 20798370

[12] Harrod CG Bendok BR Batjer HH. Prediction of cerebral vasospasm in patients presenting with aneurismal subarachnoid hemorrhage: a review. Neurosurg. (2005) 56:633–54. 10.1227/01.NEU.0000156644.45384.92 15792502

[13] Wilkins RH. Cerebral vasospasm. Crit Rev Neurobiol. (1990) 6:51–77. 2225095

[14] Akeret K Buzzi RM Schaer CA Thomson BR Vallelian F Wang S Cerebrospinal fluid hemoglobin drives subarachnoid hemorrhage-related secondary brain injury. J Cereb Blood Flow Metab. (2021) 41(11):3000–15. 10.1177/0271678X211020629 34102922 PMC8545037

[15] Chen Y Galea I Macdonald RL Wong GKC Zhang JH Rethinking the initial changes in subarachnoid haemorrhage: focusing on real-time metabolism during early brain injury. eBioMedicine. (2022) 83:104223. 10.1016/j.ebiom.2022.104223 35973388 PMC9396538

[16] Fisher CM Kistler JP Davis JM. Relation of cerebral vasospasm to subarachnoid hemorrhage visualized by computerized tomographic scanning. Neurosurgery. (1980) 6(1):1–9. 10.1227/00006123-198001000-00001 7354892

[17] Zabramski JM Spetzler RF Bonstelle C. Chronic cerebral vasospasm: effect of volume and timing of hemorrhage in a canine model. Neurosurg. (1986) 18(1):1–6. 10.1227/00006123-198601000-00001 3945370

[18] Zhang ZD Yamini B Komuro T Ono S Johns L Matron LS Vasospasm in monkeys resolves because of loss of and encasement of subarachnoid blood clot. Stroke. (2001) 32(8):1868–74. 10.1161/01.STR.32.8.1868 11486119

[19] Frontera JA Claassen J Schmidt JM Wartenberg KE Temes R Connolly ES Prediction of symptomatic vasospasm after subarachnoid hemorrhage: the modified fisher scale. Neurosurg. (2006) 59(1):21–7. 10.1227/01.neu.0000243277.86222.6c 16823296

[20] Zeineddine HA Honarpisheh P McBride DW Pandit PKT Dienel A Hong SH Targeting hemoglobin to reduce delayed cerebral ischemia after subarachnoid hemorrhage. Transl Stroke Res. (2022) 13:725–35. 10.1007/s12975-022-00995-9 35157256 PMC9375776

[21] Francoeur CL Mayer SA. Management of delayed cerebral ischemia after subarachnoid hemorrhage. Crit Care. (2016) 20:277. 10.1186/s13054-016-1447-6 27737684 PMC5064957

[22] Aralasmak A Akyuz M Ozkaynak C Sindel T Tuncer R. CT Angiography and perfusion imaging in patients with subarachnoid hemorrhage: correlation of vasospasm to perfusion abnormality. Neuroradiol. (2009) 51:85–93. 10.1007/s00234-008-0466-7 18850093

[23] Binaghi S Colleoni ML Maeder P Uske A Regli L Dehdashti AR CT Angiography and perfusion CT in cerebral vasospasm after subarachnoid hemorrhage. AJNR Am J Neuroradiol. (2007) 28:750–8. 17416833 PMC7977351

[24] Lodi CA Ursino M. Hemodynamic effect of cerebral vasospasm in humans: a modeling study. Ann Biomed Eng. (1999) 27:257–73. 10.1114/1.168 10199702

[25] Sanelli PC Ougorets I Johnson CE Riina HA Biondi A. Using CT in the diagnosis and management of patients with cerebral vasospasm. Semin Ultrasound CT MR. (2006) 7(3):194–206. 10.1053/j.sult.2006.02.004 16808218

[26] Wintermark M Ko NU Smith WS Liu S Higashida RT Dillon WP. Vasospasm after subarachnoid hemorrhage: utility of perfusion CT and CT angiography on diagnosis and management. AJNR Am J Neuroradiol. (2006) 27:26–34. 16418351 PMC7976085

[27] Dankbaar JW de Rooij NK Velthuis BK Frijns CJ Rinkel GJ van der Schaaf IC. Diagnosing delayed cerebral ischemia with different CT modalities in patients with subarachnoid hemorrhage with clinical deterioration. Stroke. (2009) 40(11):3493–8. 10.1161/STROKEAHA.109.559013 19762703

[28] Dankbaar JW Rijsdijk M van der Schaaf IC Velthuis BK Wermeret MJH Rinkel GJE Relationship between vasospasm, cerebral perfusion, and delayed cerebral ischemia after aneurysmal subarachnoid hemorrhage. Neuroradiol. (2009) 51(12):813–9. 10.1007/s00234-009-0575-y PMC2773037 19623472

[29] Rabinstein AA Friedman JA Weigand SD McClelland RL Fulgham JR Manno EM Predictors of cerebral infarction in aneurysmal subarachnoid hemorrhage. Stroke. (2004) 35(8):1862–6. 10.1161/01.STR.0000133132.76983.8e 15218156

[30] Kivisaari RP Salonen O Servo A Autti T Hernesniemi J Ohman J. MR Imaging after aneurysmal subarachnoid hemorrhage and surgery: a long-term follow-up study. AJNR Am J Neuroradiol. (2001) 22(6):1143–8. 11415911 PMC7974803

[31] Stein SC Levine JM Nagpal S LeRoux PD. Vasospasm as the sole cause of cerebral ischemia: how strong is the evidence? Neurosurg Focus. (2006) 21:E2. 10.3171/foc.2006.21.3.2 17029341

[32] Dreier JP Woitzik J Fabricius M Bathia R Major S Drenckhahn C Delayed ischaemic neurological deficits after subarachnoid haemorrhage are associated with clusters of spreading depolarizations. Brain. (2006) 129:3224–37. 10.1093/brain/awl297 17067993

[33] Dreier JP Major S Manning A Woitzik J Drenckhahn C Steinbrink J Cortical spreading ischaemia is a novel process involved in ischaemic damage in patients with aneurysmal subarachnoid haemorrhage. Brain. (2009) 132:1866–81. 10.1093/brain/awp102 19420089 PMC2702835

[34] Friedrich V Flores R Muller A Sehba FA. Luminal platelet aggregates in functional deficits in parenchymal vessels after subarachnoid hemorrhage. Brain Res. (2010) 1354:179–87. 10.1016/j.brainres.2010.07.040 20654597 PMC2933941

[35] Friedrich V Flores R Sehba FA. Cell death starts early after subarachnoid hemorrhage. Neurosci Lett. (2012) 512:6–11. 10.1016/j.neulet.2012.01.036 22306092 PMC3298610

[36] Sehba FA Mustafa G Friedrich V Bederson JB. Acute microvascular platelet aggregation after subarachnoid haemorrhage. J Neurosur. (2005) 102:1094–100. 10.3171/jns.2005.102.6.1094 16028769

[37] Budohoski KP Guilfoyle M Helmy A Huuskonen T Czosnyka M Kirollos R The pathophysiology and treatment of delayed cerebral ischaemia following subarachnoid haemorrhage. J Neurol Neurosurg Psychiatry. (2014) 85(12):1343–53. 10.1136/jnnp-2014-307711 24847164

[38] Anderson GB Ashforth R Steinke DE Findlay M. CT Angiography for the detection of cerebral vasospasm in patients with acute subarachnoid hemorrhage. Am. J Neuroradiol. (2000) 21(6):1011–5. PMC7973879 10871004

[39] Steiger HJ Ensner R Andereggen L Remonda L Berberat J Marbacher S. Hemodynamic response and clinical outcome following intravenous milrinone plus norepinephrine-based hyperdynamic hypertensive therapy in patients suffering secondary cerebral ischemia after aneurysmal subarachnoid hemorrhage. Acta Neurochir. (2022) 164:811–21. 10.1007/s00701-022-05145-6 35138488 PMC8913475

[40] American College of Radiology–American Society of Neuroradiology–Society for Pediatric Radiology (ACR-ASNR-SPR). ACR-ASNR-SPR practice parameter for the performance of computed tomography (CT) perfusion in neuroradiologic imaging. (2017). Available online at: www.asnr.org/wp-content/uploads/2019/06/CT-Perfusion-1.pdf Revised 2017.

[41] Wintermark M Lepori D Cotting J Roulet E van Melle G Meuli R Brain perfusion in children: evolution with age assessed by quantitative perfusion computed tomography. Pediatrics. (2004) 113(6):1642–52. 10.1542/peds.113.6.1642 15173485

[42] Sobesky J Zaro Weber O Lehnhardt FG Hesselmann V Thiel A Dohmen C Which time-to-peak threshold best identifies penumbral flow? A comparison of perfusion-weighted magnetic resonance imaging and positron emission tomography in acute ischemic stroke. Stroke. (2004) 35:2843–7. 10.1161/01.STR.0000147043.29399.f6 15514190

[43] Leiva-Salinas C Provenzale JM Kudo K Sasaki M Wintermark M. The alphabet soup of perfusion CT and MR imaging: terminology revisited and clarified in five questions. Neuroradiol. (2012) 54:907–18. 10.1007/s00234-012-1028-6 22488209

[44] Olivot JM Mlynash M Inoue M Marks MP Wheeler HM Kemp S Hypoperfusion intensity ratio predicts infarct progression and functional outcome in the DEFUSE 2 cohort. Stroke. (2014) 45:1018–23. 10.1161/STROKEAHA.113.003857 24595591 PMC4047639

[45] Sotoudeh H Bag AK Brooks MD. “Code-Stroke” CT perfusion; challenges and pitfalls. Acad Radiol. (2019) 26(11):1565–79. 10.1016/j.acra.2018.12.013 30655051

[46] Goldman-Yassen AE Straka M Uhouse M Dehkharghani S. Normative distribution of posterior circulation tissue time-to-maximum: effects of anatomic variation, tracer kinetics, and implications for patient selection in posterior circulation ischemic stroke. J Cereb Blood Flow Metab. (2021) 41(8):1912–23. 10.1177/0271678X20982395 33444095 PMC8327115

[47] van der Schaaf I Vonken EJ Waaijer A Velthuis B Quist M van Osch T. Influence of partial volume on venous output and arterial input function. AJNR Am J Neuroradiol. (2006) 27:46–50. 16418354 PMC7976058

[48] Kealey SM Loving VA Delong DM Eastwood JD. User-defined vascular input function curves: influence on mean perfusion parameter values and signal-to-noise ratio. Radiology. (2004) 231:587–93. 10.1148/radiol.2312030489 15064388

[49] Mangla R Ekhom S Jahromi BS Almast J Mangla M Westesson PL. CT Perfusion in acute stroke: know the mimics, potential pitfalls, artifacts, and technical errors. Emerg Radiol. (2014) 21:49–65. 10.1007/s10140-013-1125-9 23771605

[50] Konstas AA Goldmakher GV Lee TY Lev MH. Theoretic basis and technical iimplementations of CT perfusion in acute ischemic stroke. Part 1. Theoretic basis. AJNR Am J Neuroradiol. (2009) 30:662–8. 10.3174/ajnr.A1487 19270105 PMC7051780

[51] Konstas AA Goldmakher GV Lee TY Lev MH. Theoretic basis and technical implementations of CT perfusion in acute ischemic stroke. Part 2. Technical implementations. AJNR AJNR Am J Neuroradiol. (2009) 30:885–92. 10.3174/ajnr.A1492 19299489 PMC7051660

[52] Albers GW Marks MP Kemp S Christensen S Tsai JP Ortega-Gutierrez S Thrombectomy for stroke at 6 to 16 h with selection by perfusion imaging. N Engl J Med. (2018) 378:708–18. 10.1056/NEJMoa1713973 29364767 PMC6590673

[53] Nogueira RG Jadhav AP Haussen DC Bonafe A Budzik RF Bhuva P Thrombectomy 6 to 24 h after stroke with a mismatch between deficit and infarct. N Engl J Med. (2018) 378:11–21. 10.1056/NEJMoa1706442 29129157

[54] Ma H Campbell BCV Parsons MW Churilov L Levi CR Hsu C Thrombolysis guided by perfusion imaging up to 9 h after onset of stroke. N Engl J Med. (2019) 380:1795–803. 10.1056/NEJMoa1813046 31067369

[55] Campbell BCV Yassi N Ma H Sharma G Salinas S Churilv L Imaging selection in ischemic stroke: feasibility of automated CT-perfusion analysis. Int J Stroke. (2015) 10:51–4. 10.1111/ijs.123 25319251

[56] Cereda CW Christensen S Campbell BCV Mishra NK Mlynash M Levi C A benchmarking tool to evaluate computer tomography perfusion infarct core predictions against a DWI standard. J Cereb Blood Flow Metab. (2016) 36(10):1780–9. 10.1177/0271678X15610586 26661203 PMC5076783

[57] Zaro-Weber O Fleischer H Reiblich L Schuster A Moeller-Hartmann W Heiss WD. Penumbra detection in acute stroke with perfusion magnetic resonance imaging: validation with 15 O-positron emission tomography. Ann Neurol. (2019) 85:875–86. 10.1002/ana.25479 30937950 PMC6593670

[58] Schmidt JM Wartenberg KE Fernandez A Claassen J Rincon F Ostapkovich ND Frequency and clinical impact of asymptomatic cerebral infarction due to vasospasm after subarachnoid hemorrhage: clinical article. Journal of Neurosurgery JNS. (2008) 109(6):1052–9. 10.3171/JNS.2008.109.12.1052 19035719

[59] Seyour M Salvagno M Rozenblum R Macchini E Anderloni M Jodatis L The impact of perfusion computed tomography on the diagnosis and outcome of delayed cerebral ischemia after subarachnoid hemorrhage. Neurol Sci. (2024) 45:1135–44. 10.1007/s10072-023-07115-x 37828386

[60] Lagares A Cicuendez M Ramos A Salvador E Alen JF Kaen A Acute perfusion changes after spontaneous SAH: a perfusion CT study. Acta Neurochir (Wien). (2012) 154:405–11. 10.1007/s00701-011-1267-z 22234794

[61] Cremers C van der Schaaf IC Wensink E Greving JP Rinkel GJ Velthuis BK CT Perfusion and delayed cerebral ischemia in aneurysmal subarachnoid hemorrhage: a systematic review and meta-analysis. J Cereb Blood Flow Metab. (2014) 34:200–7. 10.1038/jcbfm.2013.208 24281744 PMC3915217

[62] Han H Chen Y Li R Lin F Lu J Chen X The value of early CT perfusion parameters for predicting delayed cerebral ischemia after aneurysmal subarachnoid hemorrhage: a systematic review and meta-analysis. Neurosurg Rev. (2022) 45:2517–31. 10.1007/s10143-022-01779-3v 35377027

[63] Lee DH Kang DW Ahn JS Choi CG Kim SJ Suh DC. Imaging of the ischemic penumbra in acute stroke. Korean J Radiol. (2005) 6(2):64–74. 10.3348/kjr.2005.6.2.64 15968144 PMC2686422

[64] Ditz C Hartlieb M Neumann A Machner B Schacht H Krajewski KL Routine use of perfusion computed tomography for the detection of delayed cerebral ischemia in unconscious patients after aneurysmal subarachnoid hemorrhage. Acta Neurochir (Wien). (2021) 163(1):151–60. 10.1007/s00701-020-04571-8 32910294

[65] Malinova V Tsogkas I Behme D Rohde V Psychogios MN Mielke D. Defining cutoff values for early prediction of delayed cerebral ischemia after subarachnoid hemorrhage by CT perfusion. Neurosurg Rev. (2020) 43:581–7. 10.1007/s10143-019-01082-8 30712134

[66] Sanelli PC Jou A Gold R Reichman M Greenberg E John M Using CT perfusion during the early baseline period in aneurysmal subarachnoid hemorrhage to assess for development of vasospasm. Neuroradiol. (2011) 53:425–34. 10.1007/s00234-010-0752-z PMC3118045 20694461

[67] Dong L Zhou Y Wang M Yang C Yuan Q Fang X. Whole-brain CT perfusion on admission predicts delayed cerebral ischemia following aneurysmal subarachnoid hemorrhage. Eur J Radiol. (2019) 116:165–73. 10.1016/j.ejrad.2019.05.0008 31153560

[68] Starnoni D Maduri R Hajdu SD Pierzchala K Giammattei L Rocca A Early perfusion computed tomography scan for prediction of vasospasm and delayed cerebral ischemia after aneurysmal subarachnoid hemorrhage. World Neurosurg. (2019) 130:e743–52. 10.1016/j.wneu.2019.06.213 31284055

[69] Tanabe J Nakahara I Matsumoto S Suyama Y Morioka J Oda J Cortical blood flow insufficiency scores with computed tomography perfusion can predict outcomes in aneurysmal subarachnoid hemorrhage patients: a cohort study. Neurocrit Care. (2021) 34:946–55. 10.1007/s12028-020-01108-w 33037587

[70] Lanterna LA Lunghi A Martchenko S Gritti P Bonaldi G Biroli F. Cerebral watershed hypoperfusion in subarachnoid hemorrhage: computed tomography perfusion analysis. J Neurosurg. (2011) 114:961–8. 10.3171/2010.8.JNS091766 20849218

[71] Rijsdijk M van der Schaaf IC Velthuis BK Wermer MJ Rinkel GJ. Global and focal cerebral perfusion after aneurysmal subarachnoid hemorrhage in relation with delayed cerebral ischemia. Neuroradiol. (2008) 50:813–20. 10.1007/s00234-008-0416-4 18548240

[72] van der Schaaf I Wermer M Van der Graaf Y Hoff RG Rinkel GJ Velthuis BK CT After subarachnoid hemorrhage. Relation of cerebral perfusion to delayed cerebral ischemia. Neurology. (2006) 66(10):1533–8. 10.1212/01.wnl.0000216272.67895.d3 16717213

[73] Takahashi Y Sasahara A Yamazaki K Inazuka M Kasuya H. Disturbance of CT perfusion within 24 h after onset is associated with WFNS grade but not development of DCI in patients with aneurysmal SAH. Acta Neurochir (Wien). (2017) 59(12):2319–24. 10.1007/s00701-017-3362-2 29058091

[74] Vulcu S Wagner F Santos AF Reitmeir R Söll N Schöni D Repetitive computed tomography perfusion for detection of cerebral vasospasm-related hypoperfusion in aneurysmal subarachnoid hemorrhage. World Neurosurg. (2019) 121:e739–46. 10.1016/j.wneu.2018.09.208 30308346

[75] Etminan N Beseoglu K Heiroth HJ Turowski B Steiger HJ Hänggi D. Early perfusion computerized tomography imaging as a radiographic surrogate for delayed cerebral ischemia and functional outcome after subarachnoid hemorrhage. Stroke. (2013) 44(5):1260–6. 10.1161/STROKEAHA.111.675975 23539527

[76] Rodriguez-Régent C Hafsa M Turc G Ben Hassen W Edjlali M Sermet A Early quantitative CT perfusion parameters variation for prediction of delayed cerebral ischemia following aneurysmal subarachnoid hemorrhage. Eur Radiol. (2016) 26(9):2956–63. 10.1007/s00330-015-4135-z 26670321

[77] Sanelli PC Anumula N Johnson CE Comunale JP Tsiouris AJ Riina H Evaluating CT perfusion using outcome measures of delayed cerebral ischemia in aneurysmal subarachnoid hemorrhage. AJNR Am J Neuroradiol. (2013) 34(2):292–8. https://doi.org7 10.3174/ajnr.A3225 22859289 PMC4004022

[78] Kunze E Pham M Raslan F Stetter C Lee JY Solymosi L Value of perfusion CT, transcranial Doppler sonography, and neurological examination to detect delayed vasospasm after aneurysmal subarachnoid hemorrhage. Radiol Res Pract. (2012):231206. 10.1155/2012/231206 23050146 PMC3462401

[79] Killeen RP Gupta A Delaney H Johnson CE Tsiouris AJ Comunale J Appropriate use of CT perfusion following aneurysmal subarachnoid hemorrhage: a Bayesian analysis approach. AJNR Am J Neuroradiol. (2014) 35:459–65. 10.3174/ajnr.A3767 24200901 PMC4108446

[80] Shi D Jin D Cai W Zhu Q Dou X Fan G Serial low-dose quantitative CT perfusion for the evaluation of delayed cerebral ischaemia following aneurysmal subarachnoid haemorrhage. Clin Radiol. (2020) 75(2):131–9. 10.1016/j.crad.2019.10.007 31699431

[81] Hofmann BB Fischer I Engel A Jannusch K Donaldson DM Karadag C MTT Heterogeneity in perfusion CT imaging as a predictor of outcome after aneurysmal SAH. Am. J Neuroradiol. (2021) 42(8):1387–95. 10.3174/ajnr.A7169 PMC8367610 34083263

[82] Jespersen SN Østergaard L. The roles of cerebral blood flow, capillary transit time heterogeneity and oxygen tension in brain oxygenation and metabolism. J Cereb Blood Flow Metab. (2012) 32:264–77. 10.1038/jcbfm.2011.153 22044867 PMC3272609

[83] Østergaard L Aamand R Karabegovic S Tietze A Blicher JU Mikkelsen IK The role of the microcirculation in delayed cerebral ischemia and chronic degenerative changes after subarachnoid hemorrhage. J Cereb Blood Flow Metab. (2013) 33:1825–37. 10.1038/jcbfm.2013.173 24064495 PMC3851911

[84] Duan Y Xu H Li R Zheng K Hu Z Wu N Computed tomography perfusion deficits during the baseline period in aneurysmal subarachnoid hemorrhage are predictive of delayed cerebral ischemia. J Stroke Cerebrovasc Dis. (2017 Jan) 26(1):162–8. 10.1016/j.jstrokecerebrovasdis.2016.09.004 27776892

[85] Pham M Johnson A Bartsch AJ Lindner C Müllges W Roosen K CT Perfusion predicts secondary cerebral infarction after aneurysmal subarachnoid hemorrhage. Neurology. (2007) 69:762–65. 10.1212/01.wnl.0000267641.08958.1b 17709708

[86] Laslo AM Eastwood JD Pakkiri P Chen F Lee TY. CT perfusion-derived mean transit time predicts early mortality and delayed vasospasm after experimental subarachnoid hemorrhage. Am. J Neuroradiol. (2008) 29:79–85. 10.3174/ajnr.A0747 PMC8119087 17965139

[87] Dankbaar JW de Rooij NK Rijsdijk M Velthuis BK Frjns CJM Rinkel GJ Diagnostic threshold values of cerebral perfusion measured with computed tomography for delayed cerebral ischemia after aneurysmal subarachnoid hemorrhage. Stroke. (2010) 41(9):1927–32. 10.1161/STROKEAHA.109.574392 20689085

[88] Fragata I Canhão P. Imaging predictors of outcome in acute spontaneous subarachnoid hemorrhage: a review of the literature. Acta Radiol. (2019) 60(2):247–59. 10.1177/0284185118778877 29792042

[89] Hickmann AK Langner S Kirsch M Baldauf J Müller C Khaw A The value of perfusion computed tomography in predicting clinically relevant vasospasm in patients with aneurysmal subarachnoid hemorrhage. Neurosurg Rev. (2013) 36(2):267–78 discussion 278. 10.1007/s10143-012-0430-1 23104502

[90] Murphy A Manoel AL Burgers K Kouzmina E Lee T Macdonald RL Early CT perfusion changes and blood-brain barrier permeability after aneurysmal subarachnoid hemorrhage. Neuroradiol. (2015) 57(8):767–73. 10.1007/s00234-015-1529-1 25868518

[91] Sun H Li W Ma J Liu Y You C. CT Perfusion diagnoses delayed cerebral ischemia in the early stage of the time-window after aneurysmal subarachnoid hemorrhage. J Neuroradiol. (2017) 44(5):313–8. 10.1016/j.neurad.2016.12.013 28237366

[92] van der Schaaf I Wermer MJ van der Graaf Y Velthuis BK van de Kraats CI Rinkel GJ. Prognostic value of cerebral perfusion-computed tomography in the acute stage after subarachnoid hemorrhage for the development of delayed cerebral ischemia. Stroke. (2006) 37(2):409–13. 10.1161/01.STR.0000198831.69035.43 16373646

[93] Bulsara KR Gunel M Amin-Hanjani S Chen PR Connolly ES Friedlander RM. Results of a national cerebrovascular neurosurgery survey on the management of cerebral vasospasm/delayed cerebral ischemia. J Neurointerv Surg. (2015) 7(6):408–11. 10.1136/neurintsurg-2014-011223 24811742

[94] Guenego A Fahed R Rouchaud A Walker G Faizy TD Sporns PB Diagnosis and endovascular management of vasospasm after aneurysmal subarachnoid hemorrhage—survey of real-life practices. J Neurointerv Surg. (2023) 16:677–83. 10.1136/jnis-2023-020544 37500477

[95] Hollingworth M Chen PR Goddard AJ Coulthard A Soderman M Bulsara KR. Results of an international survey on the investigation and endovascular management of cerebral vasospasm and delayed cerebral ischemia. World Neurosurg. (2015) 83(6):1120–6 e1. 10.1016/j.wneu.2015.01.036 25681601

[96] Guenego A Heit J Bonnet T Elens S Sadeghi N Ligot N Treatment of cerebral vasospasm following aneurysmal subarachnoid hemorrhage using the neurospeed non-compliant balloon. Clin Neuroradiol. (2024) 34:475–83. 10.1007/s00062-024-01390-7 38386051

[97] Rudilosso S Urra X San Román L. Perfusion deficits and mismatch in patients with acute lacunar infarcts studied with whole-brain CT perfusion. Am. J Neuroradiol. (2015) 36:1407–12. 10.3174/ajnr.A4303 PMC7964685 25882287

[98] Benson JC Payabvash S Mortazavi S Zhang L Salazar P Hoffman B CT Perfusion in acute lacunar stroke: detection capabilities based on infarct location. Am J Neuroradiol. (2016) 37:2239–44. 10.3174/ajnr 27538902 PMC7963880

[99] Cao W Yassi N Sharma G Yan B Desmond PM Davis SM Diagnosing acute lacunar infarction using CT perfusion. J Clin Neurosci. (2016) 29:70–2. 10.1016/j.jocn.2016.01.001 26899357

[100] Albers GW. Use of imaging to select patients for late window endovascular therapy. Stroke. (2018) 49:2256–60. 10.1161/STROKEAHA.118.021011 30355004

[101] Siegler JE Messé SR Sucharew H Kasner SE Mehta T Arora N Noncontrast CT versus perfusion-based core estimation in large vessel occlusion: the blood pressure after endovascular stroke therapy study. J Neuroimaging. (2020) 30(2):219–26. 10.1111/jon.12682 31762108 PMC7123741

[102] Demeestere J Wouters A Christensen S Lemmens R Lansberg MG. Review of perfusion imaging in acute ischemic stroke. From time to tissue. Stroke. (2020) 51:1017–24. https://doi-org/10.1161/STROKEAHA.119.028337 32008460

[103] Lui YW Tang ER Allmendinger AM Spektor V. Evaluation of CT perfusion in the setting of cerebral ischemia: patterns and pitfalls. Am. J Neuroradiol. (2010) 31:1552–63. 10.3174/ajnr.A2026 PMC7965002 20190208

[104] Vagal A Wintermark M Nael K Bivard A Parsons M Grossman AW Automated CT perfusion imaging for acute ischemic stroke: pearls and pitfalls for real-world use. Neurology. (2019) 93:888–98. 10.1212/WNL.0000000000008481 31636160

[105] Huang BY Castillo M. Radiological reasoning: extracranial causes of unilateral decreased brain perfusion. AJR. (2007) 189(suppl):S49–54. 10.2214/AJR.07.7030 18029901

[106] Waaijer A van der Schaaf IC Velthuis BK Quist M van Osch MJ Vonken EP Reproducibility of quantitative CT brain perfusion measurements in patients with symptomatic unilateral carotid artery stenosis. Am. J Neuroradiol. (2007) 28:927–32. PMC8134348 17494672

[107] Waaijer A van Leeuwen MS van Osch MJ van der Worp BH Moll FL Lo RT Changes in cerebral perfusion after revascularization of symptomatic carotid artery stenosis: cT measurement. Radiology. (2007) 245:541–8. 10.1148/radiol.2451061493 17848682

[108] Subhas N Primak AN Obuchowski NA Gupta A Polster JM Krauss A Iterative metal artifact reduction: evaluation and optimization of technique. Skeletal Radiol. (2014) 43:1729–35. 10.1007/s00256-014-1987-2 25172218

[109] Hakim A Slotboom J Lieger O Schlittler F Giger R Michel C Clinical evaluation of the iterative metal artefact reduction algorithm for post-operative CT examination after maxillofacial surgery. Dentomaxillofacial Radiol. (2017) 46(4):20160355. 10.1259/dmfr.20160355 PMC5595003 28112538

[110] Stidd DA Theessen H Deng Y Li Y Scholz B Rohkohl C Evaluation of a metal artifacts reduction algorithm applied to postinterventional flat panel detector CT imaging. AJNR Am J Neuroradiol. (2014) 35:2164–9. 10.3174/ajnr.A4079 25125663 PMC7965163

[111] Mennecke A Svergun S Scholz B Royalty K Dörfler A Struffert T. Evaluation of a metal artifact reduction algorithm applied to post-interventional flat detector CT in comparison to pre-treatment CT in patients with acute subarachnoid haemorrhage. Eur Radiol. (2017) 27:88–96. 10.1007/s00330-016-4351-1 27085699

[112] Bier G Bongers MN Hempel JM Örgel A Hauser TK Ernemann U Follow-up CT and CT angiography after intracranial aneurysm clipping and coiling—improved image quality by iterative metal artifact reduction. Neuroradiology. (2017) 59:649–54. 10.1007/s00234-017-1855-6 28580532

[113] Hakim A Pastore-Wapp M Vulcu S Dobrocky T Z'Graggen WJ Wagner F. Efficiency of iterative metal artifact reduction algorithm (IMAR) applied to brain volume perfusion CT in the follow-up of patients after coiling or clipping of ruptured brain aneurysms. Sci Rep. (2019) 9:19423. 10.1038/s41598-019-55792-6 31857627 PMC6923436

[114] Hakim A Vulcu S Dobrocky T Z'Graggen WJ Wagner F. Using an orbit shield during volume perfusion CT: is it useful protection or an obstacle? Clin Radiol. (2018) 73(9):834.e1–e8. 10.1016/j.crad.2018.05.003 29859633

[115] Kudo K Sasaki M Yamada K Momoshima S Utsunomiya H Shirato H Differences in CT perfusion maps generated by different commercial software: quantitative analysis by using identical source data of acute stroke patients. Radiology. (2010) 254(1):200–9. 10.1148/radiol.254082000 20032153

[116] Koopman MS Berkhemer OA Geuskens RREG Emmer BJ van Walderveen MAA Jenniskens SFM Comparison of three commonly used CT perfusion software packages in patients with acute ischemic stroke. J NeuroIntervent Surg. (2019) 11:1249–56. 10.1136/neurintsurg-2019-014822 31203208

[117] Williamson C Morgan L Klein JP. Imaging in neurocritical care practice. Semin Respir Crit Care Med. (2017) 38(6):840–52. 10.1055/s-0037-1608770 29262441

[118] Eyding J Fung C Niesen WD Krogias C. Twenty years of cerebral ultrasound perfusion imaging-is the best yet to Come? J Clin Med. (2020) 9(3):816. 10.3390/jcm9030816 32192077 PMC7141340

[119] Fung C Heiland DH Reitmeir R Niesen WD Raabe A Eyding J Ultrasound perfusion imaging for the detection of cerebral hypoperfusion after aneurysmal subarachnoid hemorrhage. Neurocrit Care. (2022) 37(1):149–59. 10.1007/s12028-022-01460-z 35211837 PMC9283360

[120] Clyde BL Resnick DK Yonas H Smith HA Kaufmann AM. The relationship of blood velocity as measured bytranscranial Doppler ultrasonography to cerebral blood flow as determinedbystablexenoncomputedtomographicstudiesafteraneurysmal subarachnoid hemorrhage. Neurosurgery. (1996) 38:896–904. 10.1097/00006123-199605000-00008 8727814

[121] Sloan MA Haley EC Jr Kassell NF Henry ML Stewart SR Beskin RR Sensitivity and specificity of transcranial Doppler ultrasonography in the diagnosis of vasospasm following subarachnoid hemorrhage. Neurology. (1989) 39:1514–18. 10.1212/WNL.39.11.1514 2682350

